# Genome-wide identification, expression profiles and regulatory network of MAPK cascade gene family in barley

**DOI:** 10.1186/s12864-019-6144-9

**Published:** 2019-10-17

**Authors:** Licao Cui, Guang Yang, Jiali Yan, Yan Pan, Xiaojun Nie

**Affiliations:** 1 State Key Laboratory of Crop Stress Biology in Arid Areas and College of Agronomy, Northwest A&F University, 3 Taicheng Road, Yangling, 712100 Shaanxi China; 2 College of Life Science, Jiangxi Agricultural University, Nanchang, 330045 Jiangxi China

**Keywords:** Barley, Gene family, MAPK cascade, Regulatory network

## Abstract

**Background:**

Mitogen-activated protein kinase (MAPK) cascade is a conserved and universal signal transduction module in organisms. Although it has been well characterized in many plants, no systematic analysis has been conducted in barley.

**Results:**

Here, we identified 20 MAPKs, 6 MAPKKs and 156 MAPKKKs in barley through a genome-wide search against the updated reference genome. Then, phylogenetic relationship, gene structure and conserved protein motifs organization of them were systematically analyzed and results supported the predictions. Gene duplication analysis revealed that segmental and tandem duplication events contributed to the expansion of barley MAPK cascade genes and the duplicated gene pairs were found to undergone strong purifying selection. Expression profiles of them were further investigated in different organs and under diverse abiotic stresses using the available 173 RNA-seq datasets, and then the tissue-specific and stress-responsive candidates were found. Finally, co-expression regulatory network of MAPK cascade genes was constructed by WGCNA tool, resulting in a complicated network composed of a total of 72 branches containing 46 HvMAPK cascade genes and 46 miRNAs.

**Conclusion:**

This study provides the targets for further functional study and also contribute to better understand the MAPK cascade regulatory network in barley and beyond.

## Background

To coordinate the biotic and abiotic stresses during growth and development, plants have evolved to form the complex mechanisms to perceive and transmit environmental stimuli by inducing or repressing a series of genes to express [1]. The Mitogen-activated protein kinase (MAPK) cascades are characterized as evolutionarily conserved and fundamentally universal signaling transduction pathways, playing the vital roles as diverse receptors/sensors from the extracellular environment to intracellular transcriptional and metabolic centers in eukaryotes [2]. The canonical MAPK cascade is composed of three specific kinases, namely MAPK, MAPK kinase (MAPKK) as well as MAPKK kinase (MAPKKK), which was activated sequentially by phosphorylation at certain activation sites [3, 4]. In general, MAPKs are phosphorylated at their conserved threonine and tyrosine residues in the activation loop (T-loop) by MAPK kinase, and in turn, MAPKK are activated by MAPKKKs when development or environmental signals incurred as their serine and serine/threonine residues located in the S/T activation site are phosphorylated [1, 2].

In plants, extensive studies have revealed that the MAPK cascades widely involved in regulating many biological processes, including cell division, plant development, growth and hormonal response as well as in response to diverse biotic and abiotic stresses, such as drought, salt, heat and pathogen infection [5–7]. In light of their importance, a large number of MAPK genes have been functionally identified in several plants, including Arabidopsis [8], rice [9–11], Brachypdoium [12, 13] and maize [14, 15]. At the same time, a series of plant MAPK signaling cascades have also been well constructed and studied. The *AtMEKK1-MKK4/5-MPK3/6* cascades is the first identified MAPK signaling module in plants, which was involved in plant innate immunity of flg22 signal transmission [16, 17]. The complete MAPK signaling cascade of ANP3-MKK6-MPK4 and YDA-MKK4/5-MPK3/6 is determined to control the stomatal development and patterning in Arabidopsis [18]. MEKK1-MKK1/2-MPK4 module was found to play the important role in the defenses against abiotic stresses and contributed to the freezing tolerance in Arabidopsis [19–21]. The ABA(abscisic acid)-activated MEKK17/18-MKK3-MPK1/2/7/14 module displayed stress signaling to ABA and regulated the expression of a series of ABA-dependent genes [22]. In tobacco, the NPK1-MEK1-Ntf6 cascade was identified to confer the resistance to tobacco mosaic virus via mediating the resistant protein N [23]. Additionally, the NPK1-NQK1/NtMEK1-NRK1 module is found to be a positive regulator of tobacco cytokinesis during meiosis as well as mitosis [24]. Barley (*Hordeum vulgare* L.) is one of the earliest domesticated and also one of the most important staple crops, which holds the significance for agriculture drawn and human civilization [25, 26]. Furthermore, barley is also well-studied in terms of cytology, genetics and genomics and thus qualifies as the model for Triticeae research [27]. The survey of MAPK family in barley has also been conducted and a total of 16 HvMAPKs were identified based on the full-length cDNA, EST(expressed sequence tag) and genomic survey database [28]. However, the incomplete data used by Krenek et al. [28] might cause the incomplete prediction and identification of MAPKK and MAPKKK family is not performed in barley up to now. The recently published reference-quality barley genome [26] makes it possible to conduct a comprehensive identification of its MAPK cascade gene families at whole genome scale and then construct the MAPK signal transduction pathway.

In this study, we systematically identified the MAPK, MAPKK and MAPKKK gene family based on a genome-wide search against barley reference genome. Then, the gene structures, chromosomal locations, gene duplication events and evolutionary dynamics were investigated. Furthermore, the expression patterns at diverse development stages and under different abiotic stresses were also analyzed. Finally, we constructed the regulatory networks of MAPK-MAPKK-MAPKKK signal pathway based on the co-expression patterns from a total of 173 RNA-seq datasets. This study reported the genomic organization, expression and phylogenetic relationships of the MAPK, MAPKK and MAPKKK gene families in barley, which could provide the candidates for further functional analysis and also contribute to illuminate the MAPK signal cascade-mediated pathway of barley and beyond.

## Results and discussion

### Genome-wide identification of MAPK cascade genes in barley

Availability of the reference-quality barley genome [26] made it possible for the first time to systematically identify all the MAPK cascade genes in this model crop species. Using the methods as described below, a total of 20 HvMAPK, 6 HvMAPKKs and 156 HvMAPKKKs were obtained, respectively (Table 1). The conserved domain analysis showed that all of them have the serine/threonine-protein kinase-like domain (PFAM accession No. PF00069) (Additional file 7: Table S1). We further validated the identified genes using the public ESTs to provide the expression support. Results showed that majority (19 out of 20 HvMAPKs, 5 out of 6 HvMAPKKs and 103 out of 156 HvMAPKKKs) of the predicted genes had the existing EST hit supports (Table 1). Given the limit of available ESTs, the non-supported HvMAPK cascade gene might not be detected under specific conditions or low levels of expression that can’t be investigated experimentally. Compared to previous study that only 16 HvMAPKs were identified by Krenek et al [28], this study found 20 HvMAPKs, which covered the 16 previous predicted ones, suggesting the whole genome-search could provide more comprehensive prediction of barley MAPK family.

**Table 1:** List of MAPK cascade genes identified in barley

No.	MAPK names	Family	Sub_Family	Ensemble barley Gene_ID	Chromosome Location	Amino acid size	EST	PI	Mw (kD)	Subcellular location	GRAGY	Ortholog
1	HvMAPK1	MAPK	–	HORVU1Hr1G049500.1	chr1H	378	3	5.75	42,892.79	Cytoplasmic	− 0.335	AtMPK4
2	HvMAPK2	MAPK	–	HORVU1Hr1G088510.1	chr1H	560	15	9.33	63,157.25	Cytoplasmic	−0.489	–
3	HvMAPK3	MAPK	–	HORVU1Hr1G090940.17	chr1H	621	11	8.38	69,944.49	Nuclear	−0.584	–
4	HvMAPK4	MAPK	–	HORVU1Hr1G091890.1	chr1H	700	21	9.55	77,086.97	Nuclear	−0.475	–
5	HvMAPK5	MAPK	–	HORVU3Hr1G056200.1	chr3H	615	23	9.04	69,867.66	Nuclear	−0.539	AtMPK20
6	HvMAPK6	MAPK	–	HORVU3Hr1G057660.34	chr3H	400	12	9.33	44,831.54	Mitochondrial	−0.336	–
7	HvMAPK7	MAPK	–	HORVU3Hr1G060390.3	chr3H	585	19	9.32	66,919.8	Nuclear	−0.546	–
8	HvMAPK8	MAPK	–	HORVU4Hr1G049430.1	chr4H	370	6	6.67	42,327.22	Nuclear	−0.18	AtMPK1
9	HvMAPK9	MAPK	–	HORVU4Hr1G057200.4	chr4H	370	10	5.46	42,811.12	Cytoplasmic	−0.299	AtMPK3
10	HvMAPK10	MAPK	–	HORVU5Hr1G078060.3	chr5H	172	–	5.02	19,465.59	Cytoplasmic	−0.028	–
11	HvMAPK11	MAPK	–	HORVU5Hr1G120960.1	chr5H	441	5	5.93	50,322.66	Cytoplasmic	−0.336	–
12	HvMAPK12	MAPK	–	HORVU6Hr1G017820.5	chr6H	213	1	7.88	24,578.45	Cytoplasmic	−0.076	–
13	HvMAPK13	MAPK	–	HORVU6Hr1G021480.1	chr6H	386	6	7.46	43,983.12	Nuclear	−0.222	–
14	HvMAPK14	MAPK	–	HORVU6Hr1G068270.1	chr6H	462	2	9.73	51,836.95	Mitochondrial	− 0.423	–
15	HvMAPK15	MAPK	–	HORVU7Hr1G008690.19	chr7H	484	9	9.44	55,037.08	Nuclear	−0.491	–
16	HvMAPK16	MAPK	–	HORVU7Hr1G023760.3	chr7H	280	7	6.33	31,878.74	Cytoplasmic	−0.116	–
17	HvMAPK17	MAPK	–	HORVU7Hr1G082510.1	chr7H	276	9	8.61	31,502.72	Cytoplasmic	0.015	–
18	HvMAPK18	MAPK	–	HORVU7Hr1G095810.7	chr7H	579	21	6.85	65,275.1	Nuclear	−0.49	AtMPK9
19	HvMAPK19	MAPK	–	HORVU7Hr1G097740.1	chr7H	370	7	7.17	42,188.13	Nuclear	−0.165	–
20	HvMAPK20	MAPK	–	HORVU0Hr1G016660.4	chrUn	462	2	9.73	51,836.95	Mitochondrial	−0.423	–
21	HvMAPKK1	MAPKK	–	HORVU1Hr1G086310.1	chr1H	331	–	9.11	35,272.53	Mitochondrial	−0.153	–
22	HvMAPKK2	MAPKK	–	HORVU5Hr1G067100.3	chr5H	233	1	8.85	26,401.41	Nuclear	−0.201	–
23	HvMAPKK3	MAPKK	–	HORVU5Hr1G125270.1	chr5H	375	2	5.92	42,093.32	Cytoplasmic	−0.214	–
24	HvMAPKK4	MAPKK	–	HORVU5Hr1G125290.3	chr5H	524	4	5.62	58,532.46	Cytoplasmic	−0.249	AtMKK3
25	HvMAPKK5	MAPKK	–	HORVU7Hr1G031720.3	chr7H	266	1	8.26	29,177.39	Mitochondrial	−0.165	AtMKK4
26	HvMAPKK6	MAPKK	–	HORVU0Hr1G038850.2	chrUn	295	1	–	–	Nuclear	−0.131	–
27	HvMEKK1	MAPKKK	MEKK	HORVU1Hr1G071060.1	chr1H	339	4	5.41	38,432.96	Cytoplasmic	−0.343	–
28	HvMEKK2	MAPKKK	MEKK	HORVU1Hr1G078710.3	chr1H	542	3	5.43	57,335.41	Chloroplast	−0.153	AtMAPKKK17
29	HvMEKK3	MAPKKK	MEKK	HORVU1Hr1G078720.3	chr1H	444	1	4.97	46,771.58	Chloroplast	−0.037	–
30	HvMEKK4	MAPKKK	MEKK	HORVU1Hr1G078760.1	chr1H	271	1	9.51	28,242.39	Chloroplast	−0.05	–
31	HvMEKK5	MAPKKK	MEKK	HORVU1Hr1G078790.1	chr1H	414	1	4.67	43,185.31	Chloroplast	−0.093	–
32	HvMEKK6	MAPKKK	MEKK	HORVU1Hr1G078860.6	chr1H	402	1	4.22	42,770.5	Cytoplasmic	−0.15	–
33	HvMEKK7	MAPKKK	MEKK	HORVU2Hr1G039070.1	chr2H	586	3	6.69	65,825.69	Cytoplasmic	−0.37	–
34	HvMEKK8	MAPKKK	MEKK	HORVU2Hr1G110900.9	chr2H	1332	6	6.05	147,411.07	Nuclear	−0.318	AtMAPKKK6
35	HvMEKK9	MAPKKK	MEKK	HORVU2Hr1G047960.2	chr2H	693	3	7	76,096.19	Nuclear	−0.671	–
36	HvMEKK10	MAPKKK	MEKK	HORVU3Hr1G065620.1	chr3H	516	2	5.14	54,444.88	Chloroplast	−0.187	AtMAPKKK16
37	HvMEKK11	MAPKKK	MEKK	HORVU3Hr1G065630.1	chr3H	470	4	5.11	49,969.2	Cytoplasmic	−0.182	–
38	HvMEKK12	MAPKKK	MEKK	HORVU3Hr1G065640.1	chr3H	490	1	5.21	52,802.78	Chloroplast	−0.286	–
39	HvMEKK13	MAPKKK	MEKK	HORVU3Hr1G087600.1	chr3H	533	4	6.65	59,672.15	Cytoplasmic	−0.433	–
40	HvMEKK14	MAPKKK	MEKK	HORVU3Hr1G109290.2	chr3H	100	1	5.36	11,266.28	Chloroplast	0.002	–
41	HvMEKK15	MAPKKK	MEKK	HORVU4Hr1G004540.4	chr4H	327	–	5.75	36,885.2	Cytoplasmic	−0.318	–
42	HvMEKK16	MAPKKK	MEKK	HORVU4Hr1G056120.2	chr4H	482	2	5.94	50,614.24	Extracellular	−0.113	AtMAPKKK14
43	HvMEKK17	MAPKKK	MEKK	HORVU4Hr1G088910.12	chr4H	741	3	6.37	81,763.31	Nuclear	−0.491	–
44	HvMEKK18	MAPKKK	MEKK	HORVU5Hr1G059030.1	chr5H	583	–	6.22	63,951.39	Chloroplast	−0.2	–
45	HvMEKK19	MAPKKK	MEKK	HORVU5Hr1G059840.4	chr5H	617	–	–	–	Nuclear	−0.566	AtMAPKKK1
46	HvMEKK20	MAPKKK	MEKK	HORVU5Hr1G094350.1	chr5H	1105	4	6.28	123,492.11	Nuclear	−0.357	–
47	HvMEKK21	MAPKKK	MEKK	HORVU5Hr1G095970.2	chr5H	837	1	5.33	90,903.5	Nuclear	−0.507	–
48	HvMEKK22	MAPKKK	MEKK	HORVU5Hr1G110900.3	chr5H	536	11	6.31	60,056.37	Cytoplasmic	−0.475	–
49	HvMEKK23	MAPKKK	MEKK	HORVU6Hr1G002500.1	chr6H	409	–	5.24	43,108.97	Chloroplast	−0.385	–
50	HvMEKK24	MAPKKK	MEKK	HORVU6Hr1G029780.1	chr6H	536	3	7.65	60,327.14	Cytoplasmic	−0.53	–
51	HvMEKK25	MAPKKK	MEKK	HORVU6Hr1G084460.23	chr6H	419	–	6.12	47,039.57	Nuclear	−0.374	–
52	HvMEKK26	MAPKKK	MEKK	HORVU7Hr1G047720.7	chr7H	703	5	6.22	78,253.71	Nuclear	−0.585	–
53	HvMEKK27	MAPKKK	MEKK	HORVU0Hr1G030360.3	chrUn	429	1	4.68	45,136.42	Chloroplast	−0.06	–
54	HvMEKK28	MAPKKK	MEKK	HORVU0Hr1G030380.1	chrUn	354	–	–	–	Chloroplast	−0.084	–
55	HvRaf-like1	MAPKKK	Raf-like	HORVU1Hr1G000090.3	chr1H	661	2	9.28	70,543.87	Nuclear	−0.43	–
56	HvRaf-like2	MAPKKK	Raf-like	HORVU1Hr1G005720.7	chr1H	763	–	6.29	85,499.18	PlasmaMembrane	−0.281	–
57	HvRaf-like3	MAPKKK	Raf-like	HORVU1Hr1G015770.2	chr1H	398	2	6.81	44,301.65	Cytoplasmic	−0.217	–
58	HvRaf-like4	MAPKKK	Raf-like	HORVU1Hr1G035440.2	chr1H	958	16	5.74	106,350.79	Nuclear	−0.475	–
59	HvRaf-like5	MAPKKK	Raf-like	HORVU1Hr1G065310.1	chr1H	445	–	5.58	49,026.03	Chloroplast	−0.216	–
60	HvRaf-like6	MAPKKK	Raf-like	HORVU1Hr1G066190.1	chr1H	857	3	6.02	92,569.39	PlasmaMembrane	−0.052	–
61	HvRaf-like7	MAPKKK	Raf-like	HORVU1Hr1G074310.4	chr1H	695	2	6.57	75,011.62	PlasmaMembrane	−0.084	–
62	HvRaf-like8	MAPKKK	Raf-like	HORVU1Hr1G075670.4	chr1H	1047	8	7.77	110,771.06	PlasmaMembrane	0.06	–
63	HvRaf-like9	MAPKKK	Raf-like	HORVU1Hr1G076110.4	chr1H	602	2	9.37	66,839.21	Nuclear	−0.492	–
64	HvRaf-like10	MAPKKK	Raf-like	HORVU1Hr1G080600.2	chr1H	635	2	5.72	70,783.85	PlasmaMembrane	−0.142	–
65	HvRaf-like11	MAPKKK	Raf-like	HORVU1Hr1G087050.1	chr1H	677	–	6.34	76,355.28	Cytoplasmic	−0.341	–
66	HvRaf-like12	MAPKKK	Raf-like	HORVU1Hr1G091230.12	chr1H	236	2	8.46	25,840.97	Cytoplasmic	−0.131	–
67	HvRaf-like13	MAPKKK	Raf-like	HORVU1Hr1G092250.3	chr1H	691	2	7.18	75,448.29	PlasmaMembrane	−0.081	–
68	HvRaf-like14	MAPKKK	Raf-like	HORVU1Hr1G092290.2	chr1H	694	–	5.77	75,163.36	PlasmaMembrane	−0.096	–
69	HvRaf-like15	MAPKKK	Raf-like	HORVU2Hr1G008140.6	chr2H	789	10	6.45	87,315.15	PlasmaMembrane	−0.199	–
70	HvRaf-like16	MAPKKK	Raf-like	HORVU2Hr1G038790.1	chr2H	694	–	6.95	77,384.09	PlasmaMembrane	−0.147	–
71	HvRaf-like17	MAPKKK	Raf-like	HORVU2Hr1G044270.3	chr2H	754	5	7.76	81,472.98	PlasmaMembrane	−0.099	–
72	HvRaf-like18	MAPKKK	Raf-like	HORVU2Hr1G044520.4	chr2H	668	6	6.8	72,527.68	PlasmaMembrane	−0.068	–
73	HvRaf-like19	MAPKKK	Raf-like	HORVU2Hr1G044590.1	chr2H	679	3	6.86	74,113.13	PlasmaMembrane	−0.159	–
74	HvRaf-like20	MAPKKK	Raf-like	HORVU2Hr1G044640.4	chr2H	254	4	5.4	28,342.25	Nuclear	−0.213	–
75	HvRaf-like21	MAPKKK	Raf-like	HORVU2Hr1G044650.1	chr2H	702	16	6.15	76,575.16	PlasmaMembrane	−0.114	–
76	HvRaf-like22	MAPKKK	Raf-like	HORVU2Hr1G044870.3	chr2H	713	2	6.75	78,483.04	Extracellular	−0.228	–
77	HvRaf-like23	MAPKKK	Raf-like	HORVU2Hr1G087930.2	chr2H	398	1	5.69	44,310.08	Cytoplasmic	−0.389	–
78	HvRaf-like24	MAPKKK	Raf-like	HORVU2Hr1G099570.11	chr2H	765	2	8.25	83,699.77	Chloroplast	−0.17	AtRaf-like6
79	HvRaf-like25	MAPKKK	Raf-like	HORVU2Hr1G104030.4	chr2H	1114	–	6	118,546.3	PlasmaMembrane	0.09	–
80	HvRaf-like26	MAPKKK	Raf-like	HORVU2Hr1G107250.1	chr2H	1005	3	6.11	109,068.87	Extracellular	0.018	–
81	HvRaf-like27	MAPKKK	Raf-like	HORVU2Hr1G123850.10	chr2H	277	–	6.22	31,174.32	Nuclear	−0.075	–
82	HvRaf-like28	MAPKKK	Raf-like	HORVU2Hr1G124370.9	chr2H	291	2	9.03	32,082.2	Mitochondrial	0.019	–
83	HvRaf-like29	MAPKKK	Raf-like	HORVU2Hr1G124530.35	chr2H	348	–	7.73	39,465.15	Nuclear	−0.395	–
84	HvRaf-like30	MAPKKK	Raf-like	HORVU2Hr1G125210.1	chr2H	666	4	8.47	73,491.31	PlasmaMembrane	−0.106	–
85	HvRaf-like31	MAPKKK	Raf-like	HORVU3Hr1G000350.4	chr3H	367	–	9.17	40,921.23	Nuclear	−0.26	–
86	HvRaf-like32	MAPKKK	Raf-like	HORVU3Hr1G000770.2	chr3H	686	1	6.39	71,693.86	Nuclear	−0.374	–
87	HvRaf-like33	MAPKKK	Raf-like	HORVU3Hr1G002820.2	chr3H	688	2	5.78	74,705.12	PlasmaMembrane	−0.066	–
88	HvRaf-like34	MAPKKK	Raf-like	HORVU3Hr1G003920.7	chr3H	401	1	6.76	43,580.58	Cytoplasmic	−0.224	–
89	HvRaf-like35	MAPKKK	Raf-like	HORVU3Hr1G006640.3	chr3H	644	5	6.25	70,581.73	PlasmaMembrane	−0.001	–
90	HvRaf-like36	MAPKKK	Raf-like	HORVU3Hr1G006790.4	chr3H	655	4	6.43	71,454.11	PlasmaMembrane	0.062	–
91	HvRaf-like37	MAPKKK	Raf-like	HORVU3Hr1G006800.2	chr3H	697	6	6.97	75,564.22	PlasmaMembrane	−0.021	–
92	HvRaf-like38	MAPKKK	Raf-like	HORVU3Hr1G017420.4	chr3H	414	3	8.84	45,791.61	Chloroplast	−0.077	–
93	HvRaf-like39	MAPKKK	Raf-like	HORVU3Hr1G026870.3	chr3H	577	2	9.59	64,845.42	Mitochondrial	−0.525	–
94	HvRaf-like40	MAPKKK	Raf-like	HORVU3Hr1G057190.5	chr3H	527	–	6.92	56,137.72	Chloroplast	−0.28	–
95	HvRaf-like41	MAPKKK	Raf-like	HORVU3Hr1G057440.1	chr3H	500	2	8.8	55,275.02	Mitochondrial	−0.416	AtRaf-like33
96	HvRaf-like42	MAPKKK	Raf-like	HORVU3Hr1G061400.1	chr3H	844	–	6.03	94,593.52	PlasmaMembrane	−0.285	–
97	HvRaf-like43	MAPKKK	Raf-like	HORVU3Hr1G061410.7	chr3H	841	–	7.54	94,745.11	PlasmaMembrane	−0.233	–
98	HvRaf-like44	MAPKKK	Raf-like	HORVU3Hr1G061450.4	chr3H	781	–	6.07	87,268.33	PlasmaMembrane	−0.142	–
99	HvRaf-like45	MAPKKK	Raf-like	HORVU3Hr1G061480.1	chr3H	835	1	6.59	91,656.76	PlasmaMembrane	−0.153	–
100	HvRaf-like46	MAPKKK	Raf-like	HORVU3Hr1G061860.2	chr3H	482	2	6.49	54,487.91	Nuclear	−0.484	AtRaf-like15
101	HvRaf-like47	MAPKKK	Raf-like	HORVU3Hr1G071240.2	chr3H	603	2	9.1	67,617.07	Nuclear	−0.593	AtRaf-like36
102	HvRaf-like48	MAPKKK	Raf-like	HORVU3Hr1G077110.18	chr3H	813	1	5.97	89,761.84	PlasmaMembrane	−0.044	–
103	HvRaf-like49	MAPKKK	Raf-like	HORVU3Hr1G077130.1	chr3H	831	1	6.08	92,032.95	PlasmaMembrane	−0.125	–
104	HvRaf-like50	MAPKKK	Raf-like	HORVU3Hr1G093140.3	chr3H	622	1	7.23	69,718.6	Nuclear	−0.504	–
105	HvRaf-like51	MAPKKK	Raf-like	HORVU3Hr1G098910.5	chr3H	308	–	5.4	35,484.92	Cytoplasmic	−0.369	–
106	HvRaf-like52	MAPKKK	Raf-like	HORVU3Hr1G109370.13	chr3H	823	12	5.84	91,309.94	PlasmaMembrane	−0.195	–
107	HvRaf-like53	MAPKKK	Raf-like	HORVU4Hr1G001850.2	chr4H	774	–	6.56	84,022.73	Chloroplast	−0.201	AtRaf-like1
108	HvRaf-like54	MAPKKK	Raf-like	HORVU4Hr1G010030.27	chr4H	875	4	8.49	95,121.28	Chloroplast	−0.234	–
109	HvRaf-like55	MAPKKK	Raf-like	HORVU4Hr1G020000.1	chr4H	374	–	6.32	40,364.96	Chloroplast	−0.094	–
110	HvRaf-like56	MAPKKK	Raf-like	HORVU4Hr1G026160.7	chr4H	844	–	5.83	95,761.31	PlasmaMembrane	−0.263	–
111	HvRaf-like57	MAPKKK	Raf-like	HORVU4Hr1G026170.1	chr4H	836	–	5.66	91,551.28	PlasmaMembrane	−0.13	–
112	HvRaf-like58	MAPKKK	Raf-like	HORVU4Hr1G026230.1	chr4H	842	–	8.45	92,169.14	PlasmaMembrane	−0.07	–
113	HvRaf-like59	MAPKKK	Raf-like	HORVU4Hr1G029350.13	chr4H	742	1	7.26	82,634.14	Nuclear	−0.596	–
114	HvRaf-like60	MAPKKK	Raf-like	HORVU4Hr1G069020.1	chr4H	392	–	6.15	43,297.77	Cytoplasmic	−0.261	–
115	HvRaf-like61	MAPKKK	Raf-like	HORVU4Hr1G069890.1	chr4H	190	–	4.64	21,123.19	Cytoplasmic	−0.236	–
116	HvRaf-like62	MAPKKK	Raf-like	HORVU4Hr1G070190.1	chr4H	396	–	5.98	43,979.3	Cytoplasmic	−0.342	–
117	HvRaf-like63	MAPKKK	Raf-like	HORVU4Hr1G073290.3	chr4H	1014	6	6.14	110,526.93	Nuclear	−0.516	AtRaf-like2
118	HvRaf-like64	MAPKKK	Raf-like	HORVU4Hr1G075550.1	chr4H	671	–	6.12	72,129.41	PlasmaMembrane	0.095	–
119	HvRaf-like65	MAPKKK	Raf-like	HORVU4Hr1G079950.13	chr4H	346	8	6.12	39,038.93	Cytoplasmic	−0.13	–
120	HvRaf-like66	MAPKKK	Raf-like	HORVU4Hr1G083590.2	chr4H	865	1	7.81	94,015.33	PlasmaMembrane	−0.082	–
121	HvRaf-like67	MAPKKK	Raf-like	HORVU4Hr1G089460.1	chr4H	113	–	4.72	12,557.55	Cytoplasmic	0.185	–
122	HvRaf-like68	MAPKKK	Raf-like	HORVU5Hr1G001800.2	chr5H	690	–	5.76	76,845.9	PlasmaMembrane	−0.292	–
123	HvRaf-like69	MAPKKK	Raf-like	HORVU5Hr1G001920.1	chr5H	1024	2	6.71	106,899.38	Chloroplast	0.134	–
124	HvRaf-like70	MAPKKK	Raf-like	HORVU5Hr1G016840.6	chr5H	1127	1	5.55	123,895.7	Nuclear	−0.484	AtRaf-like16
125	HvRaf-like71	MAPKKK	Raf-like	HORVU5Hr1G022360.3	chr5H	758	1	8.18	83,590.48	Nuclear	−0.649	AtRaf-like11
126	HvRaf-like72	MAPKKK	Raf-like	HORVU5Hr1G040040.6	chr5H	458	1	5.17	50,925.78	Cytoplasmic	−0.219	–
127	HvRaf-like73	MAPKKK	Raf-like	HORVU5Hr1G061150.2	chr5H	438	–	–	–	Nuclear	−0.473	–
128	HvRaf-like74	MAPKKK	Raf-like	HORVU5Hr1G061460.1	chr5H	388	–	5.41	43,267.93	Cytoplasmic	−0.459	–
129	HvRaf-like75	MAPKKK	Raf-like	HORVU5Hr1G077430.5	chr5H	523	–	6.27	58,965.62	Nuclear	−0.209	–
130	HvRaf-like76	MAPKKK	Raf-like	HORVU5Hr1G077450.7	chr5H	336	–	6.8	38,284.21	Cytoplasmic	−0.221	–
131	HvRaf-like77	MAPKKK	Raf-like	HORVU5Hr1G084880.1	chr5H	665	3	7.79	72,493.17	PlasmaMembrane	−0.066	–
132	HvRaf-like78	MAPKKK	Raf-like	HORVU5Hr1G085020.10	chr5H	270	1	5.39	30,562.59	Cytoplasmic	−0.311	–
133	HvRaf-like79	MAPKKK	Raf-like	HORVU5Hr1G085070.61	chr5H	555	4	5.75	60,676.75	Cytoplasmic	−0.232	–
134	HvRaf-like80	MAPKKK	Raf-like	HORVU5Hr1G089400.1	chr5H	355	2	–	–	Cytoplasmic	−0.23	–
135	HvRaf-like81	MAPKKK	Raf-like	HORVU5Hr1G093370.3	chr5H	374	4	8.52	41,309.32	Nuclear	−0.336	AtRaf-like39
136	HvRaf-like82	MAPKKK	Raf-like	HORVU5Hr1G094510.2	chr5H	389	–	6.6	42,568.27	Cytoplasmic	−0.42	–
137	HvRaf-like83	MAPKKK	Raf-like	HORVU5Hr1G095120.2	chr5H	1113	1	6.74	121,029.31	PlasmaMembrane	0.138	–
138	HvRaf-like84	MAPKKK	Raf-like	HORVU5Hr1G097010.3	chr5H	740	1	7.24	81,842.19	Nuclear	−0.414	–
139	HvRaf-like85	MAPKKK	Raf-like	HORVU5Hr1G106710.1	chr5H	249	–	7.01	27,607.12	Mitochondrial	−0.143	–
140	HvRaf-like86	MAPKKK	Raf-like	HORVU5Hr1G111670.1	chr5H	420	2	8	45,614.15	Nuclear	−0.305	AtRaf-like31
141	HvRaf-like87	MAPKKK	Raf-like	HORVU5Hr1G119060.5	chr5H	918	2	5.29	99,360.82	Nuclear	−0.441	–
142	HvRaf-like88	MAPKKK	Raf-like	HORVU5Hr1G122950.2	chr5H	1065	1	–	–	PlasmaMembrane	0.077	–
143	HvRaf-like89	MAPKKK	Raf-like	HORVU5Hr1G123540.2	chr5H	673	2	5.96	72,900.5	PlasmaMembrane	0.069	–
144	HvRaf-like90	MAPKKK	Raf-like	HORVU5Hr1G123550.1	chr5H	285	4	5.13	32,333.85	Cytoplasmic	−0.168	–
145	HvRaf-like91	MAPKKK	Raf-like	HORVU5Hr1G125710.2	chr5H	1228	3	5.37	133,759	Nuclear	−0.55	AtRaf-like20
146	HvRaf-like92	MAPKKK	Raf-like	HORVU6Hr1G012800.9	chr6H	542	–	5.83	60,241.36	Cytoplasmic	−0.304	AtRaf-like30
147	HvRaf-like93	MAPKKK	Raf-like	HORVU6Hr1G025940.2	chr6H	798	–	6.36	89,926.43	Cytoplasmic	−0.245	–
148	HvRaf-like94	MAPKKK	Raf-like	HORVU6Hr1G039740.15	chr6H	133	5	5.77	14,810.17	Extracellular	−0.048	–
149	HvRaf-like95	MAPKKK	Raf-like	HORVU6Hr1G045360.5	chr6H	429	8	8.19	48,710.48	PlasmaMembrane	−0.155	–
150	HvRaf-like96	MAPKKK	Raf-like	HORVU6Hr1G053310.1	chr6H	353	1	6.68	39,662.74	Cytoplasmic	−0.231	AtRaf-like34
151	HvRaf-like97	MAPKKK	Raf-like	HORVU6Hr1G069710.4	chr6H	422	1	8.28	46,692.96	Chloroplast	−0.128	–
152	HvRaf-like98	MAPKKK	Raf-like	HORVU6Hr1G070880.1	chr6H	820	–	6.01	92,509.74	Extracellular	−0.232	–
153	HvRaf-like99	MAPKKK	Raf-like	HORVU6Hr1G078810.22	chr6H	646	1	6.21	71,929.9	Nuclear	−0.497	–
154	HvRaf-like100	MAPKKK	Raf-like	HORVU6Hr1G083270.16	chr6H	1097	4	5.4	120,927.14	Nuclear	−0.633	AtRaf-like35
155	HvRaf-like101	MAPKKK	Raf-like	HORVU6Hr1G085710.2	chr6H	995	–	5.81	110,316.57	PlasmaMembrane	−0.005	–
156	HvRaf-like102	MAPKKK	Raf-like	HORVU6Hr1G091540.1	chr6H	465	–	9.45	49,316.59	Chloroplast	−0.323	–
157	HvRaf-like103	MAPKKK	Raf-like	HORVU7Hr1G003630.2	chr7H	433	2	6.54	48,300.04	Nuclear	−0.295	–
158	HvRaf-like104	MAPKKK	Raf-like	HORVU7Hr1G021350.1	chr7H	371	2	6.01	40,657.44	Nuclear	−0.126	–
159	HvRaf-like105	MAPKKK	Raf-like	HORVU7Hr1G029750.1	chr7H	1288	8	5.54	137,637.12	Nuclear	−0.287	AtRaf-like42
160	HvRaf-like106	MAPKKK	Raf-like	HORVU7Hr1G030370.10	chr7H	1151	1	8.24	124,540.57	PlasmaMembrane	0.059	–
161	HvRaf-like107	MAPKKK	Raf-like	HORVU7Hr1G031210.83	chr7H	823	–	6.12	90,280.99	PlasmaMembrane	−0.1	–
162	HvRaf-like108	MAPKKK	Raf-like	HORVU7Hr1G038650.5	chr7H	964	–	5.59	106,329.97	Chloroplast	−0.196	AtRaf-like4
163	HvRaf-like109	MAPKKK	Raf-like	HORVU7Hr1G041430.2	chr7H	1115	–	6.74	121,239.44	Extracellular	0.038	–
164	HvRaf-like110	MAPKKK	Raf-like	HORVU7Hr1G044510.5	chr7H	598	–	6.71	65,728.91	Cytoplasmic	−0.31	–
165	HvRaf-like111	MAPKKK	Raf-like	HORVU7Hr1G068410.1	chr7H	417	4	8.39	46,070.12	Chloroplast	−0.146	AtRaf-like28
166	HvRaf-like112	MAPKKK	Raf-like	HORVU7Hr1G078170.32	chr7H	567	–	5.63	63,786.57	Cytoplasmic	−0.343	–
167	HvRaf-like113	MAPKKK	Raf-like	HORVU7Hr1G087320.1	chr7H	548	–	6.08	62,329.43	Nuclear	−0.235	–
168	HvRaf-like114	MAPKKK	Raf-like	HORVU7Hr1G088430.1	chr7H	1055	3	5.76	114,538	PlasmaMembrane	−0.101	–
169	HvRaf-like115	MAPKKK	Raf-like	HORVU7Hr1G092030.2	chr7H	397	–	9.19	44,690.52	Nuclear	−0.359	AtRaf-like19
170	HvRaf-like116	MAPKKK	Raf-like	HORVU7Hr1G098030.1	chr7H	694	–	6.95	77,384.09	PlasmaMembrane	−0.147	–
171	HvRaf-like117	MAPKKK	Raf-like	HORVU7Hr1G109290.2	chr7H	575	1	5.37	62,356.03	Nuclear	−0.371	–
172	HvRaf-like118	MAPKKK	Raf-like	HORVU7Hr1G109640.2	chr7H	426	7	6.28	47,228.18	Chloroplast	−0.188	–
173	HvRaf-like119	MAPKKK	Raf-like	HORVU7Hr1G114620.5	chr7H	1106	9	5.63	118,989.35	Nuclear	−0.508	–
174	HvRaf-like120	MAPKKK	Raf-like	HORVU7Hr1G116190.3	chr7H	632	2	6.04	70,455.32	Cytoplasmic	−0.226	–
175	HvRaf-like121	MAPKKK	Raf-like	HORVU7Hr1G119100.1	chr7H	779	1	7.02	87,009.02	PlasmaMembrane	−0.153	–
176	HvRaf-like122	MAPKKK	Raf-like	HORVU0Hr1G011480.3	chrUn	707	2	5.97	78,559.18	PlasmaMembrane	−0.199	–
177	HvRaf-like123	MAPKKK	Raf-like	HORVU0Hr1G014630.8	chrUn	842	–	5.77	92,484.32	PlasmaMembrane	−0.107	–
178	HvRaf-like124	MAPKKK	Raf-like	HORVU0Hr1G015980.4	chrUn	397	–	8.85	43,665.02	Nuclear	−0.28	–
179	HvZIK1	MAPKKK	ZIK	HORVU2Hr1G036210.2	chr2H	352	–	6.6	39,258.55	Nuclear	−0.448	AtZIK8
180	HvZIK2	MAPKKK	ZIK	HORVU2Hr1G037990.1	chr2H	679	6	5.57	76,149.66	Nuclear	−0.515	AtZIK4
181	HvZIK3	MAPKKK	ZIK	HORVU5Hr1G046590.3	chr5H	461	1	4.91	51,074.45	Chloroplast	−0.291	AtZIK2
182	HvZIK4	MAPKKK	ZIK	HORVU6Hr1G065020.2	chr6H	619	2	4.78	69,307.43	Nuclear	−0.365	AtZIK5

Furthermore, the physical and chemical properties of these genes were investigated and compared. The length of MAPK cascade related proteins varied from 100 to 1332 amino acids, with an average of 596 in length. The putative molecular mass ranged from 11.2 kDa to 147.1 kDa, and the isoelectric points varied from 4.22 to 9.73, respectively (Table 1), which is similar to that of wheat and Brachypodium [29, 30]. The significance difference of physical and chemistry properties between the members of barley MAPK genes suggested that the subfunctionalization and neofunctionalization may have occurred among the MAPK cascade genes in barley [29]. Analysis of subcellular location showed that 52 (30%) HvMAPK cascade genes were predicted to be located in nuclear, followed by PlasmaMembrane (45) and Cytoplasmic (43), while the remaining ones were predicted to be located in chloroplast, mitochondrial and extra-cellular.

**Fig. 1:**
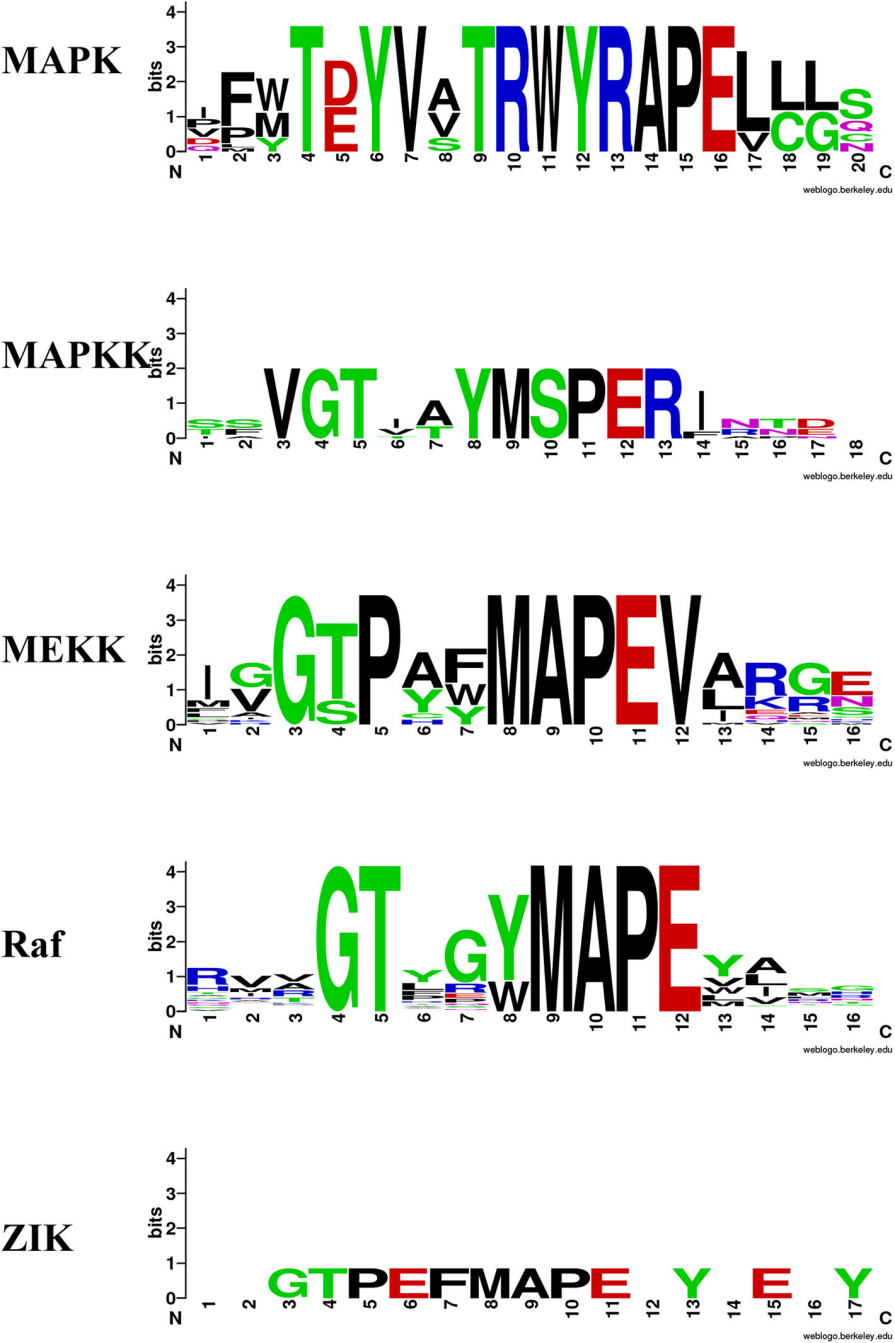
List of barley MAPK signalling components

**Fig. 2:**
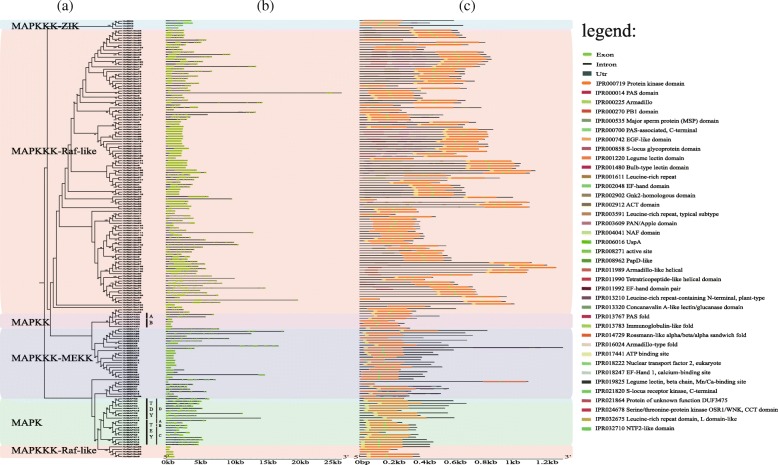
The subfamily organizations based on phylogenetic relationships (**a**), intron-exon structure structures (**b**) and protein structures (**c**) analysis of MAPK cascade genes in barley

These 182 barley MAPK cascade genes can be classified into three major clades in coordination to MAPK, MAPKK and MAPKKK with the specific conserved signature motifs, respectively (Fig. 1). Among them, 20 genes harboring the specific conserved signature motifs of T(E/D)YVxTRWYRAPE(L/V), and 6 genes possessing the VGTxxYMSPER conserved signature, which were categorized into MAPK and MAPKK subfamilies, respectively [3, 31]. Consistent with the other species [3, 10], these HvMAPKs could be assigned into the 10 TDY- and 10 TEY-subtype members (Fig. 2a and Additional file 1: Figure S1). We also investigated the docking site CD (Common docking) domain in HvMAPKs. Results showed that the TDY-subtype HvMAPKs lacked this domain (Fig. 2c and Additional file 2: Figure S2), which was the same as that of Arabidopsis [3]. All of MAPKK members contained the VGTxxYMSPER motif and the putative MAPK docking sites [K/R][K/R][K/R]x(1–5)[L/I]x[L/I] (Additional file 3: Figure S3). The remaining 156 genes belonged to MAPKKK subfamily. The barley MAPKKK genes could be further divided into three groups, which owned the conserved motifs of G(T/S)Px(W/Y/F)MAPEV, GTxx(W/Y)MAPE and GTPEFMAPE(L/V)Y for MEKK, Raf-like and ZIK subfamilies, respectively (Additional file 4: Figure S4). Remarkably, the Raf-like subfamily had 124 members, ranking the largest group of MAPKKK in barley, whereas the ZIK subfamilies possessed only 4 members as the smallest group, which was consistent with the abundance and composition of MAPKKK genes in other species, especially in wheat [29, 30] (Table 2).

**Table 2:** Comparison of the abundance of MAPK cascade gene family in different plant species

	*Hordeum vulgare*	*Triticum aestivum*	*Oryza sativa*	*Zea mays*	*Brachypodium distachyon*	*Arabidopsis thaliana*	*Lycopersicon esculentum*	*Glycine max*	*Vitis vinifera*
MAPK	20	54	17	19	16	20	16	38	14
MAPKK	6	18	8	9	12	10	6	11	5
MAPKKK	156	155	75	74	75	80	89	150	45
RAF	124	115	43	46	45	48	40	92	27
MEKK	28	29	22	22	24	21	33	34	9
ZIK	4	11	10	6	6	11	16	24	9

### Phylogenetic relationship, gene structure and motifs analysis

**Fig. 3:**
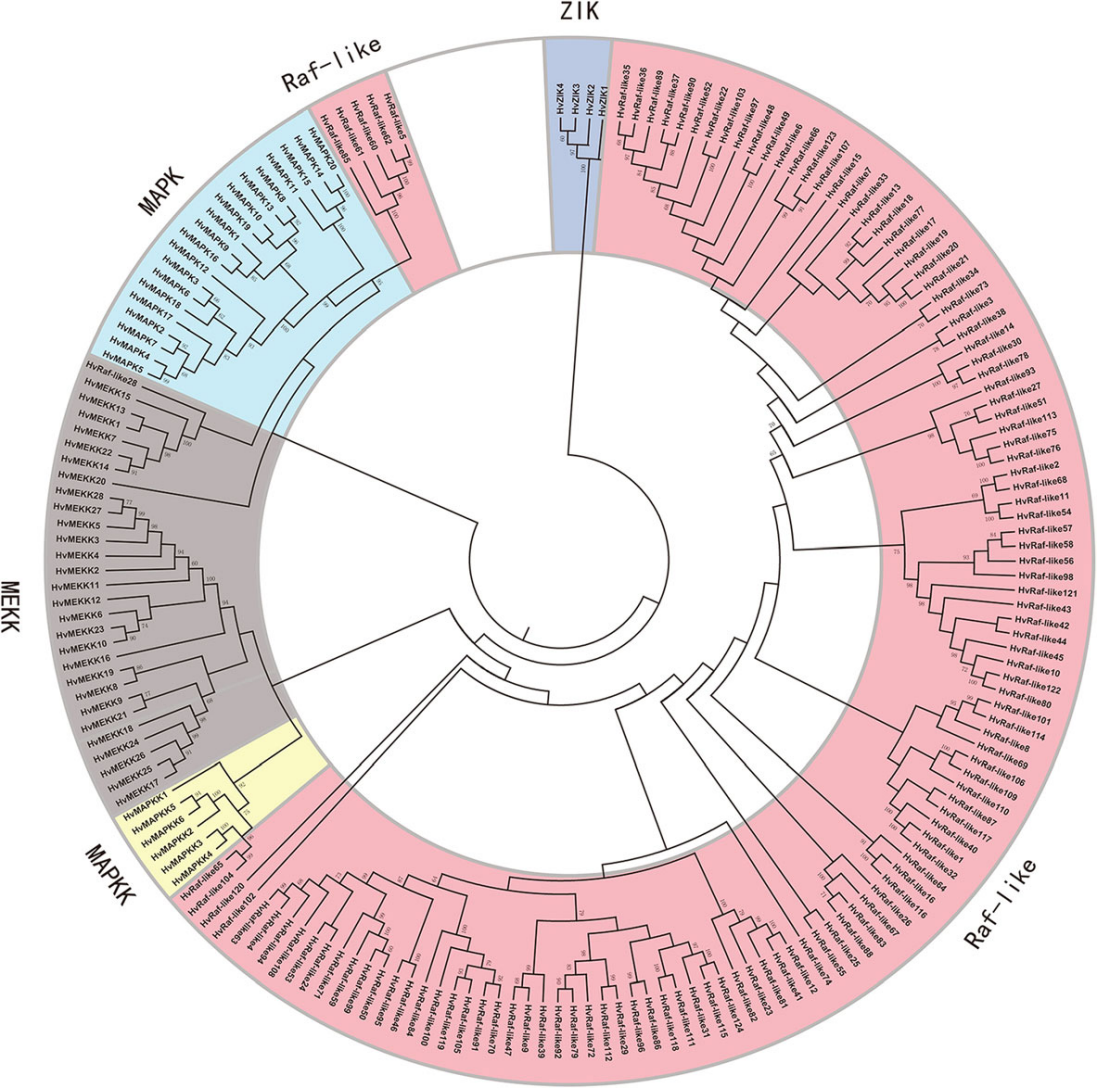
Phylogenetic analysis of barley MAPK cascade proteins

To further support the subfamily grouping, phylogenetic analysis were performed using the full-length protein sequences of these barley MAPK cascade genes (Fig. 3). Consistent with specific conserved signature motifs [3], the MEKK, Raf-like and ZIK subfamilies belonging to MAPKKK family were also clustered into independent sub-clade, respectively. For MAPK, it could be further divided into TDY and TEY two sub-clades, and TEY sub-clade was further assigned into A to C subgroups. We further performed phylogenetic analysis of these HvMAPK and the reported rice and Arabidopsis MAPKs. Results found they could clustered into different groups and the orthology pairs of them were obtained depending on phylogenetic relationship (Additional file 5: Figure S5). These results could provide some clues for candidate selection for further functional study as some orthologous genes in rice and Arabidopsis has been extensively functionally characterized [16, 18].

Gene structure played vital roles in the evolution of gene families and provided extra evidence to estimate the functional diversifications [32]. Thus, the exon-intron organization of these barley MAPK cascade genes was further analyzed (Fig. 2b). Result found that there were significant intron abundance variations between these genes. It is reported that C- and D-group of MAPKKs tend to have no introns in Arabidopsis [3]. The C-group of HvMAPKKs also showed intron-less while D-group have abundant introns. For instance, HvMAPKK3 and HvMAPKK4, which assigned into D subgroup, possessed 7 and 9 introns, respectively. Furthermore, the intron count of HvMAPKKK gene family ranged from 1 to 24, showing obviously variations even in the same subgroup. For the MEKK subfamily, more than half (54.2%) of the genes possessed no or one intron, while the other MEKK members had 6 to 24 introns. The intron number of the ZIK subfamily varied from 2 to 5, whereas the RAF genes with the intron number ranged from 1 to 20 and presented the highest level of variation among them.

Additionally, the conserved protein domains in the barley MAPK cascade genes were identified and compared. A total of 32 conserved motifs were detected (Fig. 2c). The protein kinase domain was found in each member of the MAPK cascade proteins. A certain degree of conservation could be observed in the HvMAPK and HvMAPKK genes that almost all of them harbored the ATP (Adenosine triphosphate) binding site and serine/threonine-protein kinase active site. Similar to the intron/exon structure, the composition of conserved motifs was also highly variable in HvMAPKKK family. Apart from the protein kinase and its related domains, a series of other functional motifs was widely distributed, such as Bulb-type lectin domain, S-locus glycoprotein domain and PAN/Apple domain, suggested they are widely involved in growth and development as well as signaling transduction [33]. The PAS domain, S-locus glycoprotein domain and Concanavalin A-like lectin/glucanase domain were possessed by 4, 1 and 3 Raf subfamily members. The EF-hand domain pair, EF-Hand 1, calcium-binding site and EF-hand domain were uniquely found in MEKK subfamily, whereas no domains were specific to the ZIK subfamily. On the whole, the MAPK cascade proteins clustered into the same group phylogenetically tended to share similar motifs composition.

Finally, the 1.5 kb genomic sequences upstream of the transcriptional start sites of HvMAPK genes were extracted and used to identify the cis-regulatory elements. Totally, 27 cis-elements were obtained, of which SARE(salicylic acid responsiveness) domain and the TGA(auxin-responsive) domain were found to be present only in 3 and 7 genes respectively, whereas the Skn-1 motif was shared by 159 genes, which ranked the least and most abundant motifs (Additional file 7: Table S2). Skn-1 motif is reported to be a cis-acting regulatory element required for endosperm expression and oxidative stress response in eukaryotes [34], suggesting the MAPK cascades played the important role in regulating the barley development and stress response. In addition, a large amount of plant growth and development (including circadian, meristem and endosperm), hormone-related (e.g., abscisic acid, auxin, MeJA, ethylene, gibberellin) cis-elements were found in these promoter regions, suggesting that MAPK cascade genes widely involved in regulating the signal transduction network of diverse developmental processes. Meanwhile, the cis-element related to biotic (e.g. fungal and wound) and abiotic stress response (e.g. salt, extreme temperature, dehydration) were also identified in the promoter region of the HvMAPK cascade genes, which suggested that these MAPK cascade genes might have potential functions in stress adaptation and signaling pathways [33].

### Gene duplication and synteny analysis

**Fig. 4:**
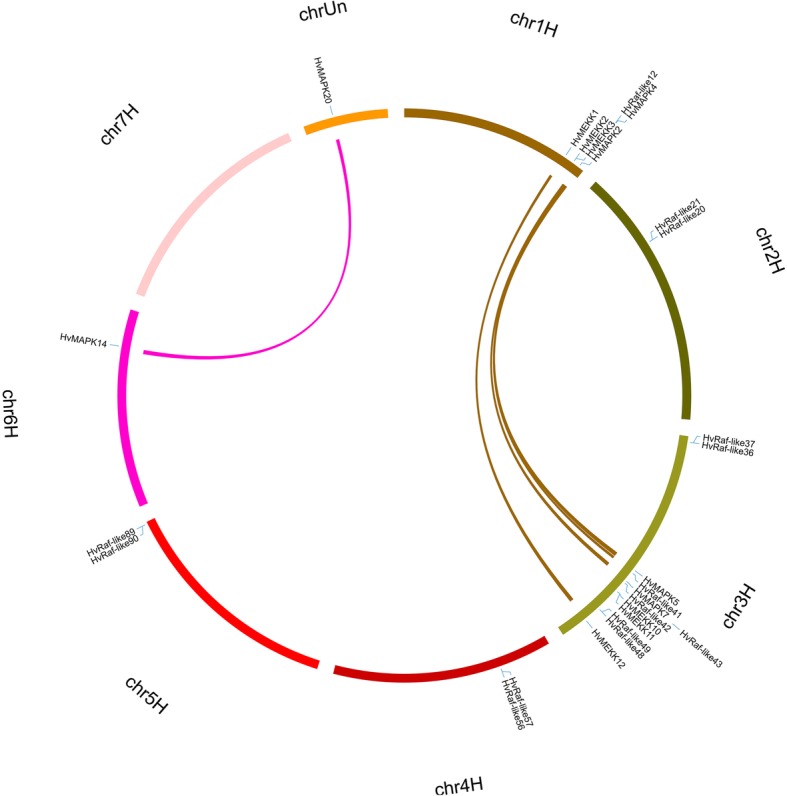
Chromosome locations and duplicated genes pairs of MAPK cascade genes in the barley genome. Each barley chromosome is displayed in different color. Duplicated gene pairs are displayed in corresponding color and linked using lines with the same color

In order to investigate the mechanism of expansion of the MAPK cascade genes in barley, we further investigated the segmental and tandem duplication events by genome synteny analysis. Results showed that 13 paralogs composed of 26 HvMAPK cascade genes were identified, of which 5 were segmental duplications and 8 were tandem duplication events (Fig. 4 and Additional file 7: Table S3). In detail, 3 and 2 segmental events were found in HvMAPKs and HvMAPKKKs, as well as 8 tandem repeats events in HvMAPKKKs, suggesting that segmental duplication played important roles in the expansion of MAPKs while tandem repeat duplication was the driven force for HvMAPKKK gene family expansion. It is noteworthy that the segmental events mainly occurred at chromosome 1 and chromosome 3, whereas the tandem duplication blocks distributed throughout the whole genome, of which 1, 1, 4, 1, 1 paralogous pairs were mapped to chromosome 1, 2, 3, 4 and 5, respectively (Fig. 4). In order to detect the selection effect during gene divergence after duplication, the Ka/Ks substitution ratio of the duplicated pairs were further calculated. Result showed that Ka/Ks ratios of MAPK cascade genes ranged from 0.001 to 0.4727, with an average of 0.1964, suggesting that they have undergone purifying selection pressure during the process of evolution in barley [35].

**Fig. 5:**
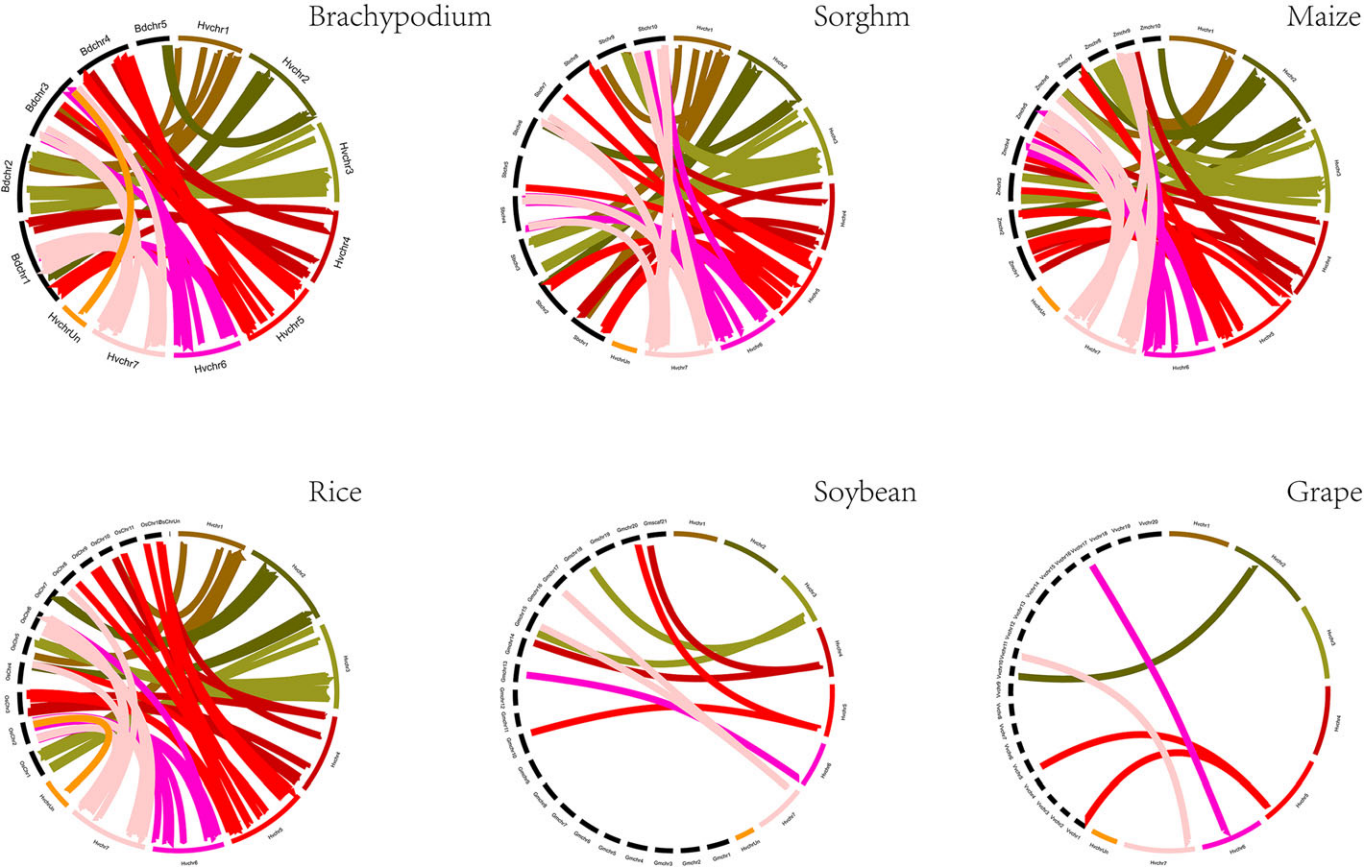
Comparative physical mapping showing the degree of orthologous relationships of MAPK cascade genes with Brachypodium, Sorghum, Maize, Rice, Soybean and Grape

Furthermore, the comparative analysis between barley with other six species (Brachypodium, sorghum, maize, rice, soybean and grape) was performed to determine the origin and evolutionary relationships of MAPK cascade genes (Fig. 5). Through whole genome-wide syntenic analysis, a total of 84, 80, 77, 67, 5 and 7 barely MAPK cascade genes were identified to have orthologous counterpart in Brachypodium, rice, sorghum, maize, grape and soybean (Additional file 7: Table S4 to S9). The average Ka/Ks value was maximum between barley and Brachypodium (0.1641), followed by rice and sorghum (0.1544) as well as maize (0.43), suggesting the genes pairs between barley and those species appeared to have undergone extensive intense purifying selection. Besides, we found that most of MAPK cascade genes showed syntenic bias towards particular chromosomes of sorghum, maize, rice, which indicated that the chromosomal rearrangement events like duplication and inversion may predominantly shape the distribution and organization of MAPK genes in these genomes [35].

### Comprehensive analysis of the expression profiles of barley MAPK cascade genes

To preliminarily predict the biological function of these barley MAPK cascade genes, gene ontology (GO) analysis was firstly performed (Additional file 6: Figure S6) and they could be annotated into 40 GO terms, including 9 terms of molecular function, 19 of biological processes and 11 of cellular components, respectively. In the cellular components category, cell and cell part were main annotation terms, whereas binding, catalytic nucleoside and transferase were the most presented function in the molecular function category. In the biological process category, cellular metabolic, cellular, metabolic and macromolecule metabolic process occupied most of the proportion. By employing the fisher statistical test method, a total of 17 terms were significant enriched (*P* < 0.05 and Q < 0.05) when taking the whole barley genome as customized backgrounds, including 5 biological process categories, 6 molecular function categories and 6 cellular component categories (Additional file 7: Table S10). These results revealed that the MAPK cascade genes played diverse roles in diverse development and stress response pathways in barley.

**Fig. 6:**
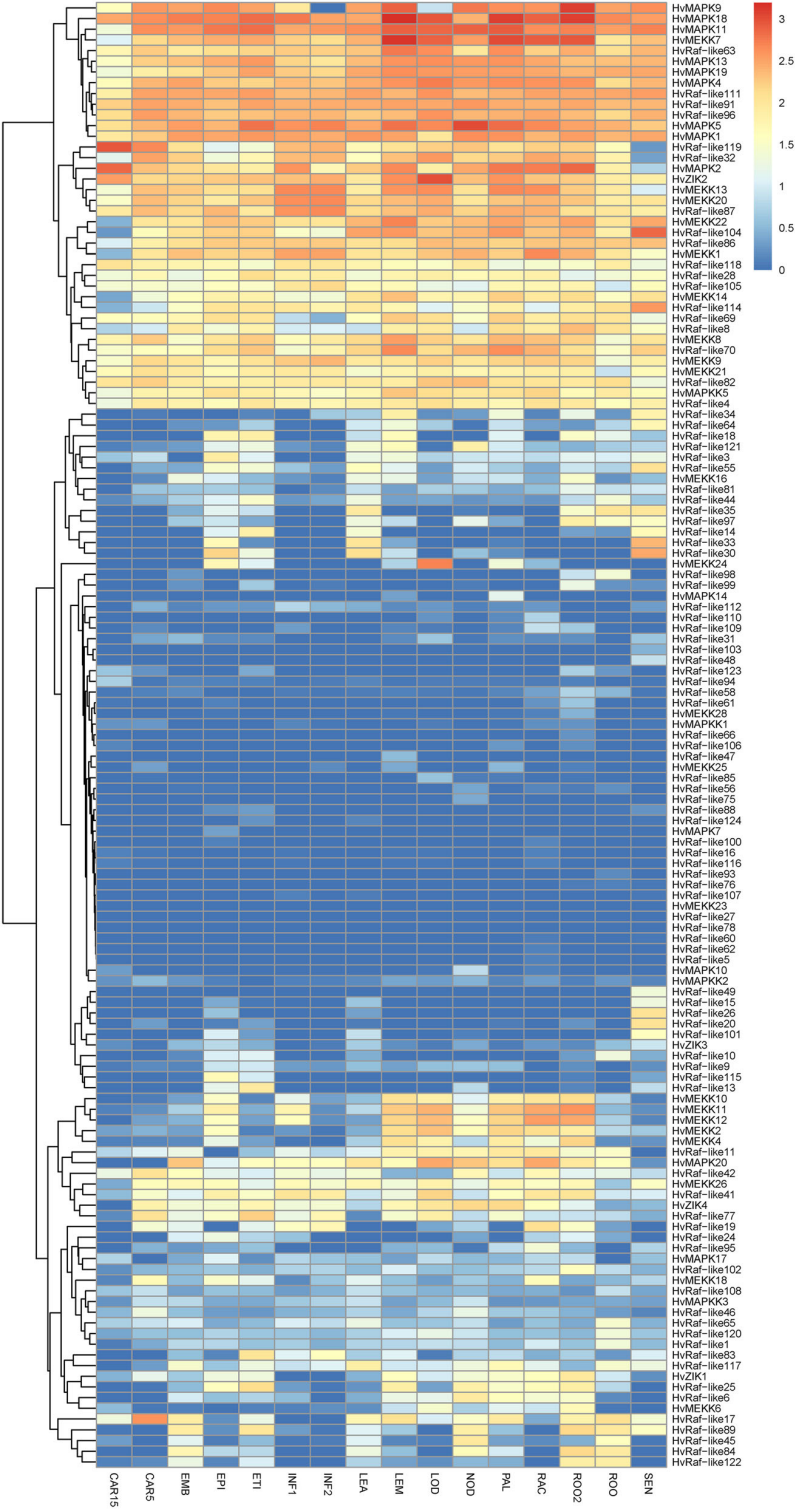
Hierarchical clustering of expression profiles of barley MAPKKK cascade genes across different stages. CAR15: bracts removed grains at 15DPA; CAR5: bracts removed grains at 5DPA; EMB: embryos dissected from 4d-old germinating grains; EPI: epidermis with 4 weeks old; ETI: etiolated from 10-day old seedling; INF1: young inflorescences with 5 mm; INF2: young inflorescences with 1–1.5 cm; LEA: shoot with the size of 10 cm from the seedlings; LEM: lemma with 6 weeks after anthesis; LOD: lodicule with 6 weeks after anthesis; NOD: developing tillers at six-leaf stage; PAL: 6-week old palea; RAC: rachis with 5 weeks after anthesis; ROO2: root from 4-week old seedlings; ROO: Roots from the seedlings at 10 cm shoot stage; SEN: senescing leaf

Furthermore, the expression profiles of MAPK cascade genes at 16 developmental stages were investigated using RNA-Seq data. A total of 75 genes were found to be expressed in at least one organ or stage (Fig. 6). A high variance in the expression levels among these MPAK cascade genes was observed, of which a series of them showed relatively high expression in all the tested tissues, such as HvMAPK1, HvMAPK4, HvRaf-like63, HvRaf-like87 and HvZIK2, The ortholog of HvZIK2 in Arabidopsis is AtZIK4(WNK1), which is found to regulating internal circadian rhythm and flowering time [36]. It highly expressed in different organs, suggesting it also played the indispensable role in organ formation and development. Additionally, the tissue- and stage-specific MAPK cascade genes were also identified. HvRaf-like103 and HvRaf-like49 were found to be predominantly expressed in senescing leaf, whereas HvRaf-like66, HvRaf-like47, HvRaf-like93 and HvMAPK7 showed preferential expression in the root, lemma, seedling root and epidermis, respectively, suggesting that these genes may mainly involve into organ- or tissue-specific development in barley.

**Fig. 7:**
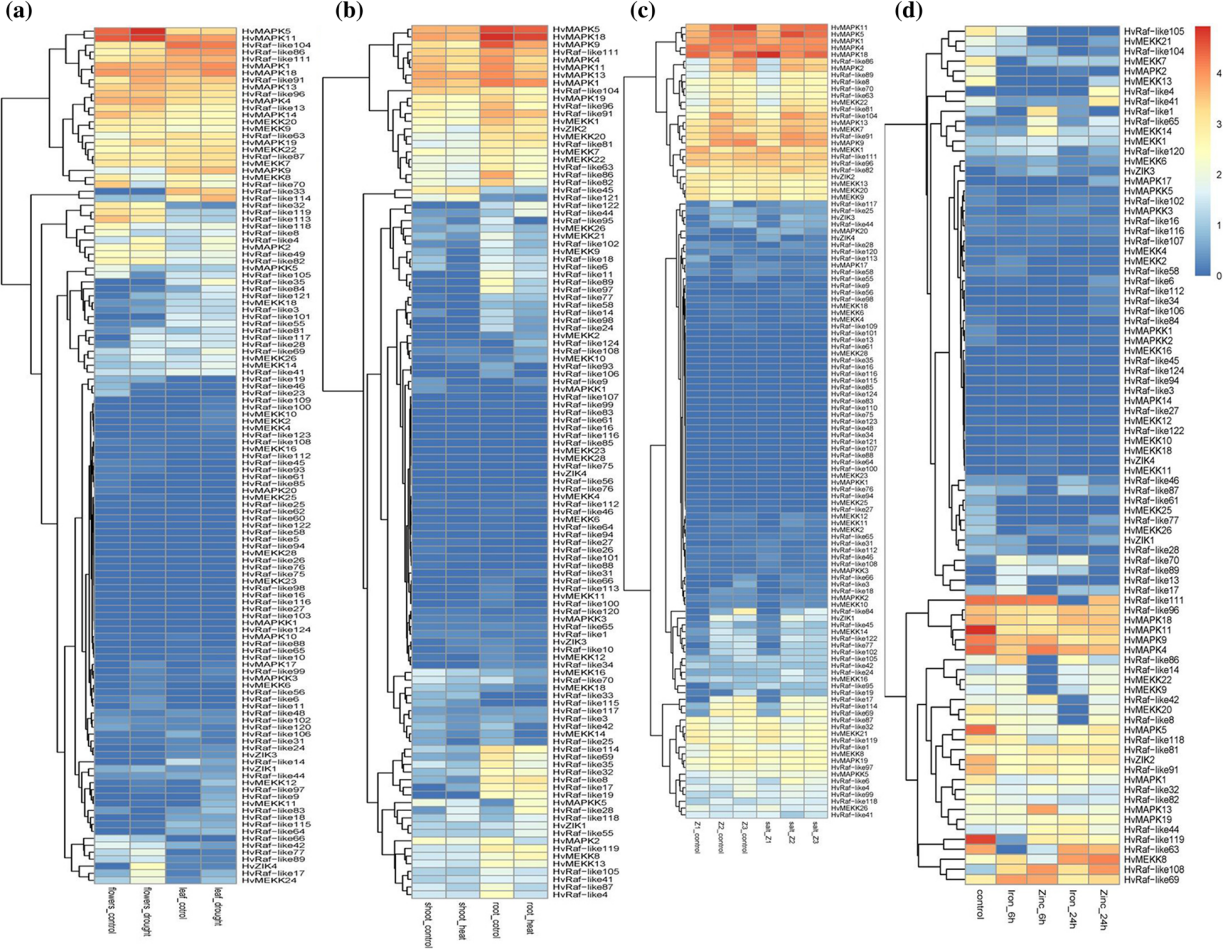
Hierarchical clustering of expression profiles of barley MAPKKK cascade genes under five stressed conditions. **a**: Drought stress; **b**: Heat stress; **c**: Salt stress; **d**: Zinc and Iron stress

To get insight into the roles of MAPK cascade genes in response to abiotic stresses, the expression profiles of them under drought, heat, salt were investigated to discover the abiotic stress-responsive candidates. Results showed that a total of 123 genes were detected to be expressed under drought stress (Fig. 7a). Among them, 10 and 24 genes were significantly up-regulated, whereas 5 and 19 MAPK cascade genes were significantly down-regulated in flowers and leaves when subjecting to drought. Meanwhile, 114 MAPK cascade genes were found to express under heat stress (Fig. 7b). Remarkably, HvRaf-like124 and HvMAPKK5 presented about 62 and 21 times higher expression level under heat stress compared to control. Previous study found the MPK20 have the defense function in cotton, while its ortholog HvMAPKK5 involved in regulating heat stress adaptation in barley, suggesting it might have divergent function in different species [37]. The expression patterns of MAPK cascades genes under salt stress were also examined (Fig. 7c). Totally, 5, 7 and 9 genes showed up-regulated in the root Z1, Z2 and Z3 respectively, of which the expression level of HvRaf-like28 and Hv-Raf-like113 were up-regulated with more than 10 fold at the Z1 zone and HvMAPKK1 showed 34-fold change at the Z2 zone. Besides, a total of 7, 11 and 4 genes were identified to be down-regulated at root Z1, Z2 and Z3 zone respectively. HvZIK4 and HvRaf-like56 was 862 and 558 time lower expression at Z1 and Z2 zone of root under salt stress than that of control.

Finally, the expression profiles of these genes under zinc metal poisoning and iron were investigated (Fig. 7d). When in response to iron stress, 9 genes showed up-regulated and 7 showed down-regulated after 6 h treatment. Furthermore, 8 up-regulated and 11 down-regulated genes were found after 24 h treatment. Among them, HvMAPK17, HvRaf-like4, HvRaf-like70, HvRaf-like109 HvZIK3and HvRafZIK4 all presented up-regulated under iron stress after both 6 h and 24 h treatment, whereas HvMAPK2 and HvRaf-like41 showed down-regulated. Under zinc stress, a total of 13 and 12 up-regulated genes as well as 14 and 16 down-regulated genes were found after 6 h and 24 h treatment, respectively. Among them HvMAPKK5, HvMEKK7, HvMEKK26, HvRaf-like28 and HvRaf-like58 were all down-regulated at all treatment, whereas HvZIK3, HvRaf-like65, HvRaf-like4, HvRaf-like108, HvMEKK14, HvMEKK10 and HvRaf-like108 displayed up-regulated after both 6 h and 24 h treatment. Obviously, HvZIK3, HvRaf-like4, HvRaf-108 showed up-regulated expression under both iron and zinc treatment, which might play the important roles in regulating signal transduction process for metal poisoning response and detoxification.

### Network construction of HvMAPK cascade genes

To get the network of miRNA targeting on MAPK cascade genes, the putative miRNAs targeted HvMAPK cascade genes were analyzed. Results found that 26 MAPK cascade genes including 3 MAPKs and 23 MAPKKK genes were predicted to be targeted by 11 miRNAs, while no miRNA target was found for HvMAPKK genes, which might be due to the limited barley miRNA reported at present (Additional file 7: Table S11). Totally, 36 miRNA-MAPK interactions were constructed based on the target relationship. The barley cascade genes were mainly inhibited by miRNAs through transcript cleavage (94.44%), while HvRaf-like12 and HvRaf-like12 and HvRaf-like76 were inhibited to translation by miRNAs. Additionally, miRNAs mainly targeted on the CDS region but behind the protein kinase domain of these MAPK cascade genes to function gene silence.

**Fig. 8:**
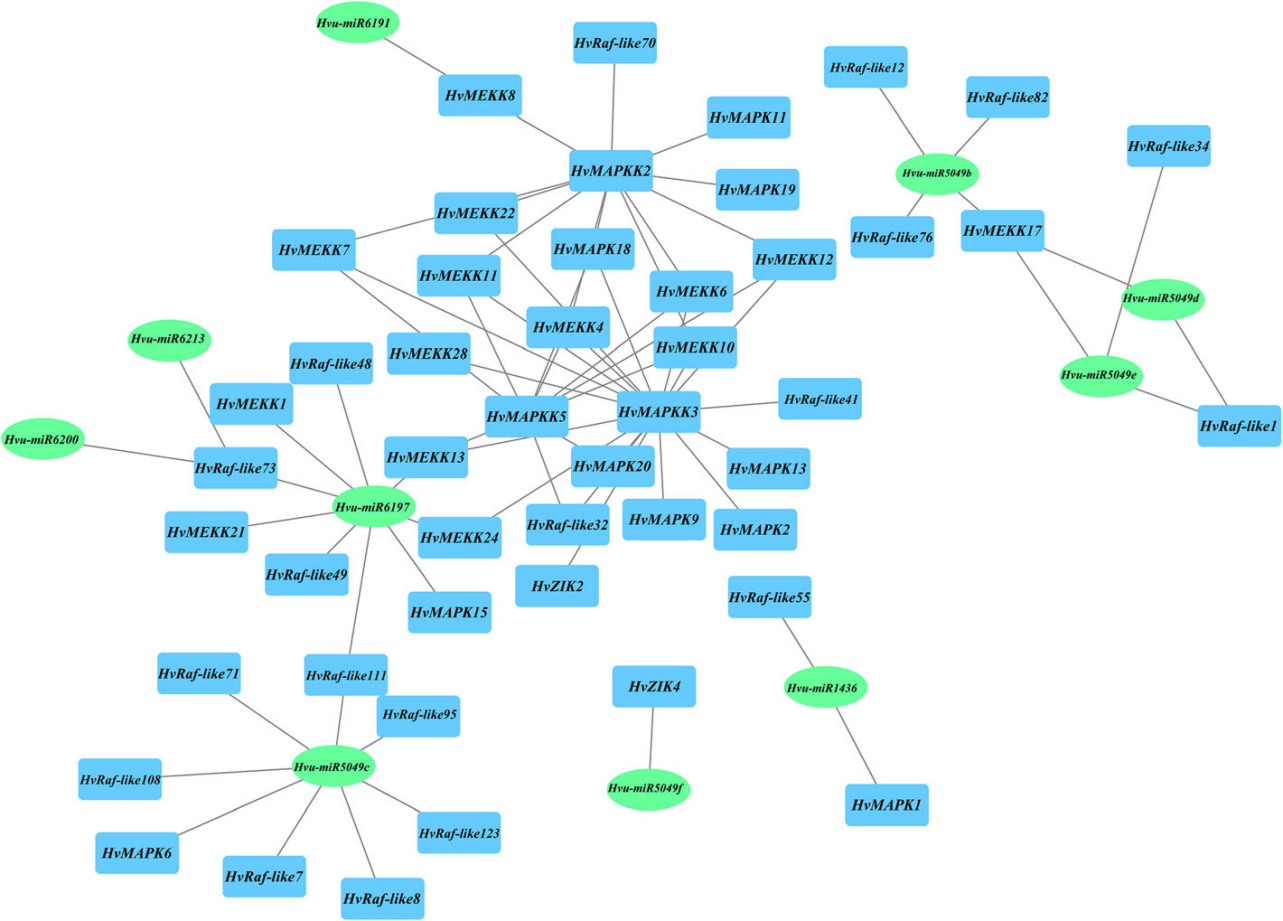
The co-expression regulatory network of MAPK cascade genes in barley. Box colour: blue, MAPK gene in barley; green, miRNA s found in barley

The co-expression regulatory network was further constructed to detect the interaction among these barley MAPK cascade genes based on weighted correlation of their expressions using a big datasets of 173 RNA-seq data. Only the relations between MAPKKK and MAPKK as well as MAPKK and MAPK were presented. A total of 40 interactions composed of 25 genes were constructed, including 7 MAPK, 3 MAPKK and 15 MAPKKK genes respectively (Fig. 8). Among them, some MAPK cascade modules has been verified in model plants, such as MKK3-MPK6 in Arabidopsis [38]and MAPK18-MAPKK2-MEKK4 in Brachypodium [30]. Furthermore, a total of 18 genes including 2 MAPK, 10 MEKK, 2 HvRaf-like and one ZIK gene were predicted to be interacted with HvMAPKK3, suggesting that it may be the hub gene of the co-expression regulatory network, playing the key role in barley MAPK cascade signaling pathway. In Arabidopsis, MAPKK3 is found to be expressed in all organs, and plays a vital role in photomorphogenesis to regulate gene expression under various light conditions, as well as involved in cell expansion, pathogen signaling and jasmonate signaling pathway, indicating it is critical for development and signaling transduction [39, 40]. Thus, the barley ortholog HvMAPKK3 might also play the hub role in co-expression network in barley response to development and stresses. In addition, there was 10 MAPK-MAPKK, 30 MAPKK-MAPKKK interactions were also obtained to use to subsequently experimental validation. Combined with miRNA-target interaction mentioned above, the regulatory network containing a total of 46 HvMAPK cascade genes and 46 miRNAs were constructed and 72 branches were linked for each other, which provided the indispensable resource to facilitate the MAPK pathway and signal transduction mechanism studies in barley and beyond.

## Conclusion

This is the first study to identify the MAPK cascade genes in barley at genomic level. Totally, 20 HvMAPKs, 6 HvMAPKKs and 156 HvMAPKKKs were obtained, which was further supported the existence by EST or full-length cDNA sequences. The phylogenetic relationships, intron-exon structure as well as conserved motif analysis all strongly supported the prediction. Furthermore, both segmental and tandem duplication events contributed to the expansion of the MAPK cascade genes in barley. The expression profiles of these MAPK cascade genes during development and under abiotic stresses were investigated and the tissue-specific or stress-responsive genes were identified, which could be considered as the candidates for further functional studies. Finally, the co-expression regulatory network of the MAPK cascade genes was constructed using WGCNA tool based on a total of 174 RNA-seq data. A total of 30 MAPKKK-MAPKK, 10 MAPKK-MAPK potential interactions were identified, which contributed to better understanding the MAPK signal transduction pathway in barely.

## Methods

### Identification of MAPK cascade genes in barley

The protein sequences of the latest updated barley genome Morex v2.0 [26] were retrieved from the IPK website (http://webblast.ipk-gatersleben.de/barley_ibsc/). Then, the MAPK cascade proteins of Arabidopsis from the TAIR database, were used as queries to search against the barley proteins using BLASTP program with an e-value of 1e-5 and identity of 50% as the threshold. The HMMER 3.0 program was employed to conduct for Hidden Markov Model (HMM) algorithm search using the serine/threonine-protein kinase-like domain (PF00069) as the query with the threshold of E < 1e− 5. The HMMER hits were further integrated with the BLASTP results and parsed by manual editing to remove redundant. Those genes displayed the consensus sequences as Jonak et al described were considered as the potential MAPK cascade genes [3]. The candidates were subsequently submitted to SMART and PFAM web tool to verify the kinase domain. Additionally, the putative MAPK cascade genes were further verified through searching against the barely ESTs by BLASTN tool. The theoretical isoelectric point (pI), molecular weight (MW) and gravy of the identified barley MAPK cascade genes were evaluated using ProtParam tool (http://web.expasy.org/protparam/) integrated in ExPASy database. The cello online server (http://cello.life.nctu.edu.tw/) was used to detect the subcellular localization and protein solubility was predicted by PROSOII tool (http://mips.helmholtz-muenchen.de/prosoII).

### Phylogenetic relationship and conserved motif analysis

Multiple sequence alignment were performed using ClustalX v2.0 with default parameter [41]. A neighbor–joining (NJ) phylogenetic tree was constructed based on the full-length protein sequences using the MEGA software with a bootstrap of 1000 replications [42]. The gene structures were obtained from the GTF annotation file of barley genome and then were displayed by Gene Structure Display Server (http://gsds.cbi.pku.edu.cn/index.php). Furthermore, the protein domain and conserved motifs of barley MAPK cascade genes were predicted using InterProScan tool. Finally, the upstream 1.5 kb genomic DNA sequences of each gene were extracted from barley genome, and then submitted to PlantCARE database to detect the putative cis-regulatory elements [43].

### Gene duplication and molecular selection analysis

Gene duplication events were defined based on the following three criteria: 1) the alignment should cover more than 70% of the longer gene; (b) the identity of the aligned region should be more than 70%; 3) for tightly linked genes only one duplication event was counted [44]. The gene synteny between barley and other species, including *Brachypodium distachyon*, *Sorghum bicolor*, *Zea mays*, *Oryza sativa*, *Vitis vinifera* and *Glycine max* was conducted using the MCScanX toolkit [45]. The linked genes pairs were displayed using the Circus tool. The rate of Ka (non-synonymous substitution)/Ks (synonymous substitution) was employed to assess the codon evolutionary rate between the synteny genes using the codeml program embedded in the PAML package [46]. The formula T = Ks/2λ was employed to calculate the duplication and divergence time, where λ referred to the mutation rate, was considered as 6.5 × 10^− 9^ synonymous substitutions per site per year.

### Expression profiles and co-expression networks construction

The MAPK cascade genes were firstly searched against the NR protein database using the local BLASTx with an E-value cut off of 10–5. Based on the Nr annotation, Blast2GO [47] program was used to retrieved the GO (gene ontology) annotation. AgriGO v2 (http://systemsbiology.cau.edu.cn/agriGOv2/index.php) was applied to conduct the singular enrichment analysis. Furthermore, a total of 172 public available RNA-seqs (Additional file 7: Table S12) including multiple tissues and developmental stages as well as biotic and abiotic stresses were downloaded from the NCBI Sequence Read Archive (http://www.ncbi.nlm.nih.gov/sra) database to investigate the expression profiles of these genes. The FPKM (fragments per kilobase of transcript per million fragments mapped reads) value were calculated by Hisat2 and Stringtie software [48]. Then, differentially expressed genes were identified with the following threshold values: fold change≥2, FDR(false discovery rate) ≤ 0.01, and the absolute ratio of log2 ≥ 1. All FPKM data was finally reported by log2 counts and the heat map was visualized using pheatmap package in R. WGCNA was used to construct the co-expression network based on all of the downloaded transcriptome data [49]. Besides, all the identified MAPK cascade transcripts were submitted to the psRNATarget tool [50] to search the barley miRNAs targets in the miRBase. The regulatory network of Hvu-miRNA and HvMAPK cascade genes were visualized using cytoscape tool (http://www.cytoscape.org/).

## Supplementary information

**Additional file 1: Figure S1.** Multiple sequence alignment of the partial sequences of 20 HvMAPK proteins to identify the TDY and TEY motif. The red color marked sequence is the TDY or TEY motif.

**Additional file 2: Figure S2.** Multiple sequence alignment of the full length sequence of 20 HvMAPK proteins to identify the conserved kinase motifs. The color marked indicated the conserved motifs found.

**Additional file 3: Figure S3.** Multiple sequence alignment of the HvMAPKK to identify the conserved kinase motifs. The red color marked are the signature motif of MAPKK proteins.

**Additional file 4: Figure S4.** Multiple sequence alignment of the HvMAPKKK to identify the conserved kinase motifs. The red color marked are the signature motif of MEKK, Raf and ZIK three sub family.

**Additional file 5: Figure S5.** Evolutionary relationships and grouping among barley, rice and Arabidopsis MAPKs.

**Additional file 6: Figure S6.** GO annotation of these identified barley MAPK cascade genes.

**Additional file 7: Table S1.** Motif identification based on PFAM database. **Table S2.** Characteristics of cis-acting regulatory elements in the promoter region of these identified barley MAPK cascade genes. **Table S3.** Duplicated MAPK cascade gene pairs identified in barley. **Table S4.** The Ka/Ks ratios for orthologous MAPK cascade proteins between barley and brachypodium. **Table S5.** The Ka/Ks ratios for orthologous HvMAPK cascade proteins between barley and rice sorghum. **Table S6.** The Ka/Ks ratios for orthologous MAPK cascade proteins between barley and maize. **Table S7.** The Ka/Ks ratios for orthologous MAPK cascade proteins between barley and sorghum. **Table S8.** The Ka/Ks ratios for orthologous MAPK cascade proteins between barley and soybean. **Table S9.** The Ka/Ks ratios for orthologous MAPK cascade proteins between barley and grape. **Table S10.** GO annotation of the identified barley MAPK cascade genes. **Table S11.** List of the putative miRNAs targeted on HvMAPK cascade genes identified by psRNATarget online tool. **Table S12.** Accession number and sample information of RNA-seq data using in this study.

## Data Availability

The datasets supporting the conclusions of this article are included within the article and its additional files.

## Abbreviations

ABA: Abscisic acid
ATP: Adenosine triphosphate
CD: Common docking
EST: Expressed sequence tag
FDR: False discovery rate
FPKM: Fragments per kilobase of transcript per million fragments mapped reads
GO: Gene ontology
HMM: Hidden Markov Model
Ka: Non-synonymous substitution
Ks: Synonymous substitution
MAPK/MPK: Mitogen-activated protein kinase
MAPKK/MKK: Mitogen-activated protein kinase kinase
MAPKKK/MEKK: Mitogen-activated protein kinase kinase
MeJA: Methyl jasmonate
miRNA: MicroRNA
MW: Molecular weight
PAS: Per-Arnt-Sim
pI: Isoelectric point
SARE: Salicylic acid responsiveness
TDY: Putative activation motif in MAPK gene TDY1
TGA: Auxin-responsive element
