# Supplementary material for: Genome-wide identification, expression profiles and regulatory network of MAPK cascade gene family in barley
Source: BMC Genomics. 2019 Oct 17;20:750. doi: 10.1186/s12864-019-6144-9 (PMC6796406; doi:10.1186/s12864-019-6144-9)
Supplement: Supplementary file 1 — Additional file 1: Figure S1. Multiple sequence alignment of the partial sequences of 20 HvMAPK proteins to identify the TDY and TEY motif. The red color marked sequence is the TDY or TEY motif. [file 12864_2019_6144_MOESM1_ESM.pdf]

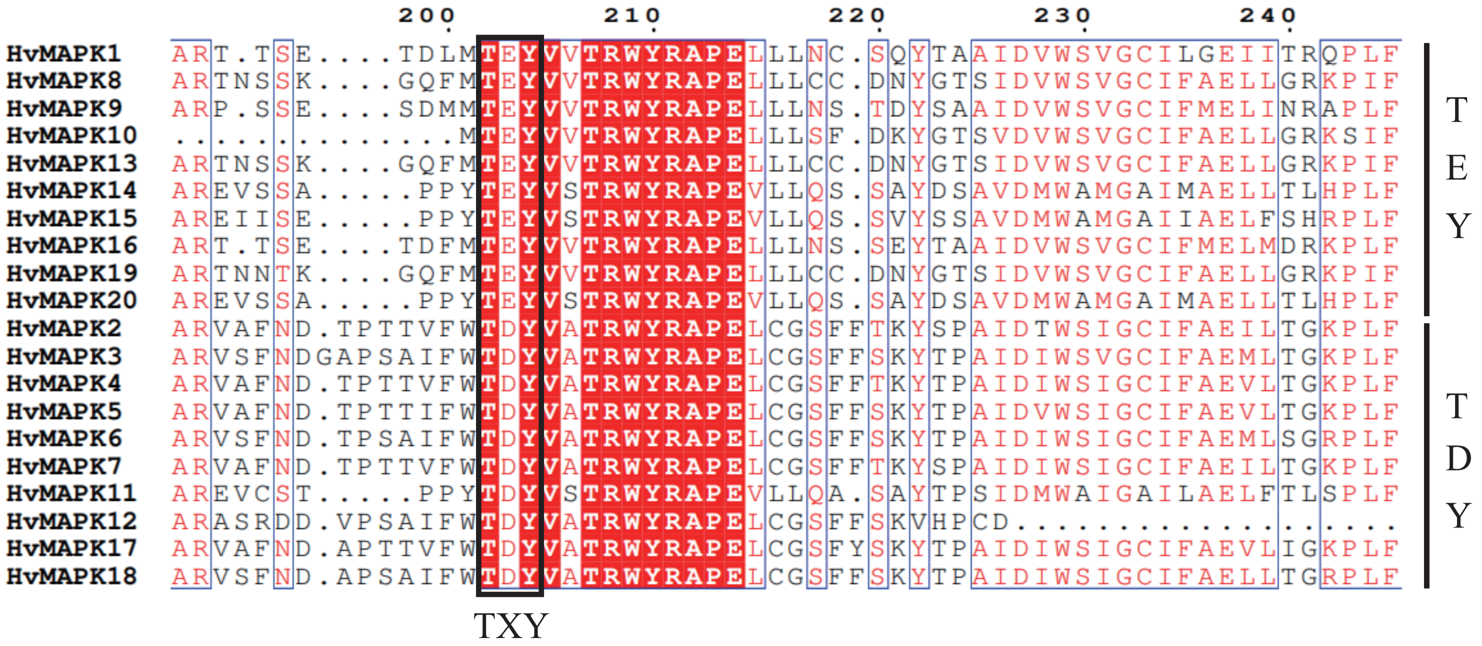
200
210
220
230
240
HvMAPK1
HvMAPK8
HvMAPK9
HvMAPK10
HvMAPK13
HvMAPK14
HvMAPK15
HvMAPK16
HvMAPK19
HvMAPK20
HvMAPK2
HvMAPK3
HvMAPK4
HvMAPK5
HvMAPK6
HvMAPK7
HvMAPK11
HvMAPK12
HvMAPK17
HvMAPK18
TEY
TDY
TXY
