# Supplementary material for: Genome-wide identification, expression profiles and regulatory network of MAPK cascade gene family in barley
Source: BMC Genomics. 2019 Oct 17;20:750. doi: 10.1186/s12864-019-6144-9 (PMC6796406; doi:10.1186/s12864-019-6144-9)
Supplement: Supplementary file 2 — Additional file 2: Figure S2. Multiple sequence alignment of the full length sequence of 20 HvMAPK proteins to identify the conserved kinase motifs. The color marked indicated the conserved motifs found. [file 12864_2019_6144_MOESM2_ESM.pdf]

```
HvMAPK2    ............................................................
HvMAPK7    ............................................................
HvMAPK4    .QSRQEKEIYLPPPPPLPSGIHPLSGAPPDRARRGGRAPTLPLSSPAAACARSPRRVRLA
HvMAPK5    ............................................................
HvMAPK17   ............................................................
HvMAPK18   .MGGGNGIVDG....................FRRLFHRRTPSGSVLGSSNQSSAGEDSSE
HvMAPK3    PPPNAPAHPRNRQRCMGGRARSLIRWLRHHRSRRVSSASSSSSSHLTHTNTSSATTATSD
HvMAPK6    PIARLPLHPR.......................RVHAP.......LPHPRPSVPAEARHT
HvMAPK12   ............................................................
HvMAPK9    ............................................................
HvMAPK16   ............................................................
HvMAPK1    ............................................................
HvMAPK10   ............................................................
HvMAPK19   ............................................................
HvMAPK8    ............................................................
HvMAPK13   ............................................................
HvMAPK11   ............................................................
HvMAPK15   ............................................................
HvMAPK20   ............................................................
HvMAPK14   ............................................................

                                                             1
HvMAPK2    ......................................................MPEANA
HvMAPK7    ......................................................MQPDQQ
HvMAPK4    VRESGGPAP................................RAGGQGSVRSPEPSWRADR
HvMAPK5    ............................................................
HvMAPK17   ............................................................
HvMAPK18   L........................................EAVEDLDLVGLRPIRVPKR
HvMAPK3    LRARSLPLQQDEDDDVEAGWEEEQDEFAEGPESDPEGYIVLEREGDAGSLRVVVPRAPAR
HvMAPK6    ARTRAP...................EPVEG..VDPGG.....SETARGSTSLRSTGRLAR
HvMAPK12   .........................................LFLLDYHLVTLG.......
HvMAPK9    .....................................................MDGAPVA
HvMAPK16   .....................................................APGGAME
HvMAPK1    .............................................MDTSGGGGGAAGGAA
HvMAPK10   ............................................................
HvMAPK19   ......................................................MAMMVD
HvMAPK8    ......................................................MAMLVD
HvMAPK13   ......................................PSSPRPSCPPSPSHLKMAMLVD
HvMAPK11   ............................................................
HvMAPK15   ............................................................
HvMAPK20   ............................................................
HvMAPK14   ............................................................

              10        20        30        40        50        60
HvMAPK2    GARGGGEQRSKSSDVMSFFSEYGDASRYKI.EEIGKGSYGVVCSAIDRQTGDKVAIKKI
HvMAPK7    ....QHQQRRKGSSDMDFFSEYGDANRYKI.QEVIGKGSYGVVCSAIDQHNGDKVAIKKI
HvMAPK4    PVRQPVMQRSKSSAEVDFFTEYGDANRYKI.QEVIGKGSYGVVCSALDLQTRQKVAIKKI
HvMAPK5    ...MQQDQRKKSSAEAEFFTEYGDASRYKI.QEIVGKGSYGVVCSAIDVHTGEKVAIKKI
HvMAPK17   ..MQNIDLRKKSAAEIDFFTEYGDTNRYKV.LEVIGKGSYGLVCSANDTQTGEKVAIKKI
HvMAPK18   KMPLPVESHKKNIMEKEFFTEYGEASQYQI.QEVVGKGSYGVVAAAIDTRTGERVAIKKI
HvMAPK3    TKPPPRMDPGKKTSESEFFTEYGELNRYQV.SEVIGKGSYGVVAAAIDTQTGERVAIKKI
HvMAPK6    RAPCLKRRGG..SDKAGFFTEYGEATRYEV.GEVVGKGSYGVVAAAVDTHTGERVAIKKI
HvMAPK12   ...............MEFFTEYGEASQYQI.KEIIGKGSFGVVAAAVDTQTGEWVAIKKI
HvMAPK9    EFRPTMTHGGRFLLYNIFGNQFEITAKYQPPIMPIGRGAYGIVCSVMNFETREMVAIKKI
HvMAPK16   NIQATLSHGGRFIQYNIFGNVFEVTAKYKPPILPIGKGAYGIVCSALNSETGEQVAIKKI
HvMAPK1    QIQGMATHGGRYVLYNVYGNLFEVASKYAPPIRPIGRGAYGIVCAAVSSDTGEEVAIKKI
HvMAPK10   ............................................................
HvMAPK19   PPNGIGNHGK..HYYTMWQTMFEIDTKYVP.IKPIGRGAYGIVCSSINQETNEKVAIKKI
HvMAPK8    PPNGMGNQGK..HYYSMWQTLFEIDTKYVP.IKPIGRGAYGIVCSSINRETNEKVAIKKI
HvMAPK13   PPNGMGNQGK..HYYSMWQTLFEIDTKYVP.IKPIGRGAYGIVCSSINRETNEKVAIKKI
HvMAPK11   ........................MERYKV.IREIGDGTCGNVFRAYNIETNEIVAVKKM
HvMAPK15   ........................MERYNI.ITEVGDGTFGSVWRAINKESGEVVAIKKM
HvMAPK20   ........................MDRFKL.IKEVGDGTFGSVWRAINKQNGEVVAVKKM
HvMAPK14   ........................MDRFKL.IKEVGDGTFGSVWRAINKQNGEVVAVKKM

               70        80        90       100       110       120
HvMAPK2    SNIFEHITDAARILREIKLLRLLRHPDIVQIKHIMLPPSRRDFKDIFVVFELMDTDLHQV
HvMAPK7    HNIFEHLSDAARILREIKLLRLLRHPDIVEIRHIMLPPSRRDFKDIYVVFELMDTDLHQV
HvMAPK4    HNIFEHTSDAARILREIKLLRLLRHPDVVEIKHIMLPPSRKDFKDIYVVFELMESDLHQV
HvMAPK5    HDIFEHISDAARILREIKLLRLLRHPDIVEIKHIMLPPSRRDFKDIYVVFELMESDLHQV
HvMAPK17   HNIFEHISDAARILREIKLLRLLRHPDVVEIKHILLPPSKKDFKDIYVVFELMESDLHQV
HvMAPK18   NDVFEHVSDATRILREVKLLRLLRHPDVVEIKHIMLPPSRREFQDIYVVFELMESDLHQV
HvMAPK3    NDVFDHVSDATRILREIKLLRLLRHPDIVQIKHIMLPPSRREFRDIYVVFELMESDLHQV
HvMAPK6    DDVFEHVSDATRILREVKLLRLLRHPDIVQIKHIMLPPSRREFRDIYIVFELMESDLHQV
HvMAPK12   HDMFEHASDGTRILREIKLLRLLRHPNIVEIKHILLPPARREFRDIYVVFELMESDLQKV
HvMAPK9    ANAFDNNMDAKRTLREIKLLKHLDHENIVGLRDVIPPAIPQSFNDVYIATELMDTDLHHI
HvMAPK16   ANAFDNKIDAKRTLREIKLLRHMDHENIVAIRDIIPPAQRTAFNDVYIAYELMDTDLHQI
HvMAPK1    GNAFDNHIDAKRTLREIKLLRHMDHENILAMKDLIRPPRRDDFKDVYIVTELMDTDLHQI
HvMAPK10   ............................................................
HvMAPK19   NNVFDNRVDALRTLRELKLLRHLRHENVIALKDIMMPIHRRSFKDVYLVSELMDTDLHQI
HvMAPK8    HNVFDNRVDALRTLRELKLLRHLRHENVISLKDIMMPVQRRSFKDVYLVYELMDTDLHQI
HvMAPK13   HNVFDNRVDALRTLRELKLLRHLRHENVISLKDIMMPVQRRSFKDVYLVYELMDTDLHQI
HvMAPK11   KRKFYHWEECIS.LREVKALQKLNHPNIVQLKEVTM.....ENHELFFIFEHMDCNLYDV
HvMAPK15   KKKYFSWEECIN.LREVKSLRRMNHPNIVKLKEVIR.....ENDMLFFVFEYMECNLYQL
HvMAPK20   KRKYYSFEECMS.LREVKSLRRMNHPNIVKLKEVIR.....ENDILYFIMEYMECNLYQL
HvMAPK14   KRKYYSFEECMS.LREVKSLRRMNHPNIVKLKEVIR.....ENDILYFIMEYMECNLYQL
```

```
                130       140       150       160       170       180
HvMAPK2     IKAN.DDLTKEHFQFFLYQMLRAMKYIHTANVYHRDLKPKNILANANCKLKICDFGLARV
HvMAPK7     IKAN.DDLTKEHHQFFLYQMLRALKYIHTANVYHRDLKPKNILANANCKLKICDFGLARV
HvMAPK4     IKAN.DDLTKEHYQFFLYQLLRALKYIHTASVYHRDLKPKNILANSNCKLKICDFGLARV
HvMAPK5     IKAN.DDLTKEHYQFFLYQLLRALKYIHTANVYHRDLKPKNILANSNCKLKICDFGLARV
HvMAPK17    IKAN.DDLTREHYQFFLYQMLRALKYMHTANVYHRDLKPKNVLANANCKLKICDFGLARV
HvMAPK18    IRAN.DDLTAEHYQFFLYQLLRALKYIHGANVFHRDLKPKNILANADCKLKICDFGLARV
HvMAPK3     IKAN.DDLTPEHHQFFLYQLLRGMKYIHAASVFHRDLKPKNILANADCKLKICDFGLARV
HvMAPK6     IKAN.DDLSPEHHQFFFYQLLRGMKYIHAANVFHRDLKPKNILANADCKLKICDFGLARV
HvMAPK12    IQVN.DNLTKGHHRFFLYQLLHALKYIHTANVFHRDLKPSNILANSNCKLKICDFGLARA
HvMAPK9     IRSN.QELSEEHCQYFLYQLLRGLKYIHSANVIHRDLKPSNLLLNANCDLKICDFGLARP
HvMAPK16    IRSN.QALSEEHCQYFLYQILRGLKYIHSANVLHRDLKPSNLLLNANCDLKICDFGLART
HvMAPK1     IRSN.QSLTDDHCQYFLYQLLRGLKYVHSANVLHRDLKPSNLFLNANCDLKIADFGLART
HvMAPK10    ............................................................
HvMAPK19    VKSS.QPLSNDHCQYFLFQLLRGLKYLHSAGILHRDLKPGNLLVNANCDLKICDFGLART
HvMAPK8     IKSP.QGLSNDHCQYFLFQLLRGLKYLHSAEILHRDLKPGNLLVNANCDLKICDFGLART
HvMAPK13    IKSP.QGLSNDHCQYFLFQLLRGLKYLHSAEILHRDLKPGNLLVNANCDLKICDFGLART
HvMAPK11    IRERSAPFSEEEIRKFMLQILQGLVYMHNNGYFHRDLKPENLLVSN.GIVKIADFGLARE
HvMAPK15    MKSKGKPFSETEIRNWCFQVFQALSHMHQRGYFHRDLKPENLLVTK.ELIKVADFGLARE
HvMAPK20    MKDRVKPFAESDVRNWCFQIFQALAYMHQRGYFHRDLKPENLLVSK.DVLKLADFGLARE
HvMAPK14    MKDRVKPFAESDVRNWCFQIFQALAYMHQRGYFHRDLKPENLLVSK.DVLKLADFGLARE
```

```
                190       200       210       220       230       240
HvMAPK2     AFND.TPTTVFWTDYVATRWYRAPELCGSFFTKYSPAIDTWSIGCIFAEILTGKPLFPGK
HvMAPK7     AFND.TPTTVFWTDYVATRWYRAPELCGSFFTKYSPAIDIWSIGCIFAEILTGKPLFPGK
HvMAPK4     AFND.TPTTVFWTDYVATRWYRAPELCGSFFTKYTPAIDIWSIGCIFAEVLTGKPLFPGK
HvMAPK5     AFND.TPTTIFWTDYVATRWYRAPELCGSFFSKYTPAIDVWSIGCIFAEVLTGKPLFPGK
HvMAPK17    AFND.APTTVFWTDYVATRWYRAPELCGSFYSKYTPAIDIWSIGCIFAEVLIGKPLFPGK
HvMAPK18    SFND.APSAIFWTDYVATRWYRAPELCGSFFSKYTPAIDIWSIGCIFAELLTGRPLFPGK
HvMAPK3     SFNDGAPSAIFWTDYVATRWYRAPELCGSFFSKYTPAIDIWSVGCIFAEMLTGKPLFPGK
HvMAPK6     SFND.TPSAIFWTDYVATRWYRAPELCGSFFSKYTPAIDIWSIGCIFAEMLSGRPLFPGK
HvMAPK12    SRDD.VPSAIFWTDYVATRWYRAPELCGSFFSKVHPCD......................
HvMAPK9     .SSE....SDMMTEYVVTRWYRAPELLLNS.TDYSAAIDVWSVGCIFMELINRAPLFPGR
HvMAPK16    .TSE....TDFMTEYVVTRWYRAPELLLNS.SEYTAAIDVWSVGCIFMELMDRKPLFPGR
HvMAPK1     .TSE....TDLMTEYVVTRWYRAPELLLNC.SQYTAAIDVWSVGCILGEIITRQPLFPGR
HvMAPK10    ...........MTEYVVTRWYRAPELLLSF.DKYGTSVDVWSVGCIFAELLGRKSIFPGT
HvMAPK19    NNTK....GQFMTEYVVTRWYRAPELLLCC.DNYGTSIDVWSVGCIFAELLGRKPIFPGT
HvMAPK8     NSSK....GQFMTEYVVTRWYRAPELLLCC.DNYGTSIDVWSVGCIFAELLGRKPIFPGT
HvMAPK13    NSSK....GQFMTEYVVTRWYRAPELLLCC.DNYGTSIDVWSVGCIFAELLGRKPIFPGT
HvMAPK11    VCST.....PPYTDYVSTRWYRAPEVLLQA.SAYTPSIDMWAIGAILAELFTLSPLFPGE
HvMAPK15    IISE.....PPYTEYVSTRWYRAPEVLLQS.SVYSSAVDMWAMGAIIAELFSHRPLFPGS
HvMAPK20    VSSA.....PPYTEYVSTRWYRAPEVLLQS.SAYDSAVDMWAMGAIMAELLTLHPLFPGT
HvMAPK14    VSSA.....PPYTEYVSTRWYRAPEVLLQS.SAYDSAVDMWAMGAIMAELLTLHPLFPGT
```

```
                250       260       270       280       290       300
HvMAPK2     NVVHQLDLMTDFLGSPSPDIISRIRNEKARRYLSTMRKKLPVPFSEKFPNADPAAVKLLQ
HvMAPK7     NVVHQLDLMTDLLGTPSLDTVSRIRNEKARRYLSSMRKKQTVCFSERFPKADPAALKLMQ
HvMAPK4     NVVHQLDLMTDLLGTPSMDTISRVRNEKARRYLTSMRKKDPVPFSQKFPNADPLGVKLLE
HvMAPK5     NVVHQLDLMTDLLGTPSMDTISRVRNEKARRYLTSMRKKEPISFSHKFPNADPLALDLLQ
HvMAPK17    NVVHQLDLITDVLGTPSLDAISQVWIFGG........................FAVKVIV
HvMAPK18    NVVHQLDIITDLLGTPSSETLSRIRNEKARRYLSCMRKKHPVPLTQKFPNADPLAVRLLG
HvMAPK3     NVVHQLDLMTDVLGTPSAESLAKIRNEKARRYLSNMRKKPKVPLTKKFPGIDPMALHLLE
HvMAPK6     NVVHQLDLMTDLLGTPSAESISRIRNEKARRYLGNMKKKHPIPFSQKFPGVDPMALHLLE
HvMAPK12    ............................................................
HvMAPK9     DHMHQMRLITEVIGTPTDDDLGFIRNEDARRYMRHLPQFPRRPFPGQFPKVQPAALDLIE
HvMAPK16    DHVHQLRLLMELISTPSVPKYSLFR.DSTTDYIQMY...................IDIL.
HvMAPK1     DYIQQLKLITELIGSPDDSSLGFLRSDNARRYMKQLPQYPRQDFRLRFRNMSDGAVDLLE
HvMAPK10    DCLNQLKLIVDVLGTMSDANLEFIGNTKARKYVKSLSYTVGVPLTRMYPQAHPLAIDLLE
HvMAPK19    ECLNQLKLIVNVLGTMSEADLAFIDNSKARKYIKSLPYTPGIPLSSMYPQAHPLAIDLLQ
HvMAPK8     ECLNQLKLIVNVLGTMSESDLEFIDNPKARRYIKTLPYTPGVPLASMYPHAHPLAIDLLQ
HvMAPK13    ECLNQLKLIVNVLGTMSESDLEFIDNPKARRYIKTLPYTPGVPLASMYPHAHPLAIDLLQ
HvMAPK11    TETDQLFKICAVLGTPD.HSLWPEGMNLPRSSSFQFFQIPPRNLWELIPNASLEALDLIK
HvMAPK15    SEADEIYKICNILGTPN.QHTWAGGLQLAASIHFQFPQSGSINLSEVVPTASEDALNLIS
HvMAPK20    SEADEILKICNVIGSPD.EQTWPQGLSLAEAMKYQFPQIRGNQLSEVMKSASSEAVDLIS
HvMAPK14    SEADEILKICNVIGSPD.EQTWPQGLSLAEAMKYQFPQIRGNQLSEVMKSASSEAVDLIS
```

```
                310       320             330       340       350
HvMAPK2     KLLAFDPKDRPTAEEALADPYFKG....LAKVEREPSCQPISKMEFEFERRKFTKEEVKE
HvMAPK7     RLLAFDPKDRPTAEEALADPYFKG....LGKVEREPSCQPISKFEFEFERKKVTKEDVKE
HvMAPK4     KLLAFDPKDRPTAEEALTDPYFRG....LSKPEREPSCQPIRKMEFDFEHSRVSKDDIRE
HvMAPK5     RLLAFDPKDRPTAEEALSHPYFKG....LAKVEREPSCQPITKMEFEFERRRVTKEDIRE
HvMAPK17    RVCL........................................................
HvMAPK18    RLLAFDPKDRPSAEEALADPYFAS....LANVEREPSRHPISKLEFEFERRKVTKDDVRE
HvMAPK3     RLLAFDPKDRPTADEALTDPYFTG....LANSEREPIAQPISKLEFEFEKRKLGKDDVRE
HvMAPK6     RLLAFDPADRPTAAEALADPYFTG....LANSELEPTTQPISKT................
HvMAPK12    ............................................................
HvMAPK9     RMLTFNPLQRITVEEALEHPYLER....LHDVADEPICT..DPFSFDFEQHPLTEDQMKQ
HvMAPK16    ............................................................
HvMAPK1     RMLVFDPSRRITVDEALHHPYLAS....LHDINEEPTCP..APFSFDFEQPSFTEEHMKE
HvMAPK10    KMLVFDPSKRISVTEALEHPYMST....LYDPSANHPAQ..APIDLDIDEK.LGVDMIRE
HvMAPK19    KMLVFDPSKRISVTQALEHPYMSP....LYDPSANPPAQ..VPIDLDIDEN.IGTDMIRE
HvMAPK8     KMLIFDPTKRISVTQALEHPYMSP....LYDPSANPPAQ..VPIDLDIDEN.ISSEMIRE
HvMAPK13    KMLIFDPTKRISVTQALEHPYMSP....LYDPSANPPAQ..VPIDLDIDEN.ISSEMIRE
HvMAPK11    QLCSWDPRRRPTAEQALQHPFFNV.RKWVQRPLHDASYSKMNEPRATPPRLELD.....L
HvMAPK15    WLCSWDPRKRPTAVEVLQHPFFQQPCFYVPPSLRYRSTGYATPPPSVGAKGAMDQKNARR
HvMAPK20    SLCSWDPCKRPKAAEVLQHAFFKD.CTYVPASVRPKVAGPPKTPPGVGVRGVSG.HIARR
HvMAPK14    SLCSWDPCKRPKAAEVLQHAFFKD.CTYVPASVRPKVAGPPKTPPGVGVRGVSG.HIARR
```

```
             360       370       380       390       400       410
HvMAPK2   LIFREILEYHPQLLKDYTNGSEKTNFLYPSAVDNFRRQFANLEEDGGKGGAPE...RKHV
HvMAPK7   LIFREILEYHPQLLKDYMNGTEKTNFLYPSAVDNFRRQFANLEENGGKGGAVVPSDRKHV
HvMAPK4   LIFQEILEYHPQLLKSYIDGTERTTFLYPSAVDHFKKQFSHLEESDGSG.PVVPADRKHA
HvMAPK5   LIFREILEYHPQLLKDYTNGTERTTFLYPSAVDQFRKQFAHLEENTENG.PVVPMDRKHT
HvMAPK17  ............................................................
HvMAPK18  LIYREILEYHPQMLEEYMKGGDQISFLYPSGVDRFKRQFAHLEEHYSKGERGSPLQRKHA
HvMAPK3   LIYREILEYHPHMLQEYLRGGDQMSFMYPSGVDRFKQQFAHLEKGGAKGDKSSPQLRQNA
HvMAPK6   ............................................................
HvMAPK12  ............................................................
HvMAPK9   LIFNEALELNPNFRY.............................................
HvMAPK16  ............................................................
HvMAPK1   LIWRETLAFNPDPPY.............................................
HvMAPK10  MLWQEMLQYP..................................................
HvMAPK19  MLWQEMLQYHPEAARMVNM.........................................
HvMAPK8   MMWQEMLHYHPEAAAAVNM.........................................
HvMAPK13  MMWQEMLHYHPEAAAAVNM.........................................
HvMAPK11  WGFGTESDD.LDLTLSLKPS..SVSDLGKR........VPE.....RREEQNLLYPGYG
HvMAPK15  YPVGTLSNGRPAVNNSYLSTNAPARAAGVQRKLELDHQVKPEGNHKLTTKENAMNQPWSR
HvMAPK20  YSTGALSTSKPAGNISIKPN..SLSKIGVQRKLQLDRQA.PE.KKSTRPAESNTKQTTSR
HvMAPK14  YSTGALSTSKPAGNISIKPN..SLSKIGVQRKLQLDRQA.PE.KKSTRPAESNTKQTTSR
```

```
                420       430                                     440
HvMAPK2   SLPRTTTVHSTPIPTTSGP...........................ASQAPQRIPTA
HvMAPK7   SLPRTTTVHSTPIPPKDQK...........................PSQVPQRIPTG
HvMAPK4   SLPRSTIVHSTPIPAKDTR...PLFGKPCS....KTSSETGRYAGNGHGASQASHAAQAV
HvMAPK5   SLPRSTIVHSAPIHAKEQPRIGPSRDKPSPDESYKNPRDSEKYSGNVQRTSLAPQRVPTA
HvMAPK17  ............................................................
HvMAPK18  SLPRQRVGASNDSNNEQHI...................................SDQEMS
HvMAPK3   SLPRERAIGNKHGDSEYQV...................................KLN..T
HvMAPK6   ............................................................
HvMAPK12  ............................................................
HvMAPK9   ............................................................
HvMAPK16  ............................................................
HvMAPK1   ............................................................
HvMAPK10  ............................................................
HvMAPK19  ............................................................
HvMAPK8   ............................................................
HvMAPK13  ............................................................
HvMAPK11  NPP..VQPGLWPLMSSSHRP........................................
HvMAPK15  LPPAPVRNNMNYLAAKEQIPR...........................GGAPDIA
HvMAPK20  VPARNSPVNANPMLRHSRS.........................................
HvMAPK14  VPARNSPVNANPMLRHSRS.........................................
```

```
                450       460       470         480       490       500
HvMAPK2   RPGRVVASATPIENAAFADRQTGRRMS..RDPAAPPAAAAAGYTLRPDCPDRQQQQQQQE
HvMAPK7   RPGRVVGPVIPFENSCAMDPYSQRRVA..RNPVLPAAATNLSAYAYHRKSDNTERELQQE
HvMAPK4   VSRRAAGSALPYEGGSGKHPYD.VASRPAMSTGCPPQQQIPQMYGQYQHQAPAGAGAGAG
HvMAPK5   RPGRVVGPVLPYENGGMKDTYDPRRVAAAMNSGYAPQQQIPQIYGYYQTPAKSACSEPSQ
HvMAPK17  ............................................................
HvMAPK18  AEPDAHGAVSPQKPQDAPGVGQNGLSPTSLSSRTYLKSASISASKCVVVNPNKQPEYDDA
HvMAPK3   GEKPVHASVT...........DGISKPLMSARSLLKSETMSASKCIGEIKNKDDEL...
HvMAPK6   ............................................................
HvMAPK12  ............................................................
HvMAPK9   ............................................................
HvMAPK16  ............................................................
HvMAPK1   ............................................................
HvMAPK10  ............................................................
HvMAPK19  ............................................................
HvMAPK8   ............................................................
HvMAPK13  ............................................................
HvMAPK11  ..LGEAPVMPSWQQAYMLDSHATTLPAAAGGGGFPGSSPFGLFPLQ.....PNLMENRSL
HvMAPK15  EKLSQLSMSTTTRAPIMPSDRFVDLKATTRAHGEPVRRPVPLGPRDTWHARNDPFRRTYE
HvMAPK20  ..LPETGRATVHKVSSIT.ERLAHMSVTSRTR.TTVKPPVPLLKAG..HLKSDFLGKSDE
HvMAPK14  ..LPETGRATVHKVSSIT.ERLAHMSVTSRTR.TTVKPPVPLLKAG..HLKSDFLGKSDE
```

```
                  510       520         530
HvMAPK2   LEKDRTRYRPALHFRDARVAPEA.EARPSAYYIP..........................
HvMAPK7   LEKDRMQYQPMQRFMDAKMVSP..DLRSSSYYMPKGVPKADVAERTALQPNMMQG.....
HvMAPK4   AGIP.QAMGGYACGGYAKGTAPAPAMRAPTYRHVPAGQKNGRLDRLAVETTDIYTRSLNG
HvMAPK5   AEMYTLHQQAYACANSSTVPDVALDMRAPPFHQS.AVPKRGSSDRLSAES.NLYSRSLNG
HvMAPK17  ............................................................
HvMAPK18  ISEETEGAVDGLSEKVSKMHA.......................................
HvMAPK3   ...ESVDAADGVSQKIAQLKT.......................................
HvMAPK6   ............................................................
HvMAPK12  ............................................................
HvMAPK9   ............................................................
HvMAPK16  ............................................................
HvMAPK1   ............................................................
HvMAPK10  ............................................................
HvMAPK19  ............................................................
HvMAPK8   ............................................................
HvMAPK13  ............................................................
HvMAPK11  AP..APIRQVNFF...............................................
HvMAPK15  MPGERALLQRKLVS..............................................
HvMAPK20  IP.PAKRLTRKLVS..............................................
HvMAPK14  IP.PAKRLTRKLVS..............................................
```

```
                 540          550
                   .            .
HvMAPK2   ...PFNGIAAVAGGYSKVGAA....ARMY
HvMAPK7   .IAPFNGIAAVGGSYNKASAVQYGVSRMY
HvMAPK4   IVAAAAASAGTASAHRKVGTVPFGMPTTY
HvMAPK5   ..MAPTTSGVAASGHRKVGVVPYGVSQMY
HvMAPK17  .............................
HvMAPK18  .............................
HvMAPK3   .............................
HvMAPK6   .............................
HvMAPK12  .............................
HvMAPK9   .............................
HvMAPK16  .............................
HvMAPK1   .............................
HvMAPK10  .............................
HvMAPK19  .............................
HvMAPK8   .............................
HvMAPK13  .............................
HvMAPK11  .............................
HvMAPK15  .............................
HvMAPK20  .............................
HvMAPK14  .............................
```
