# Supplementary material for: Genome-wide identification, expression profiles and regulatory network of MAPK cascade gene family in barley
Source: BMC Genomics. 2019 Oct 17;20:750. doi: 10.1186/s12864-019-6144-9 (PMC6796406; doi:10.1186/s12864-019-6144-9)
Supplement: Supplementary file 3 — Additional file 3: Figure S3. Multiple sequence alignment of the HvMAPKK to identify the conserved kinase motifs. The red color marked are the signature motif of MAPKK proteins. [file 12864_2019_6144_MOESM3_ESM.pdf]

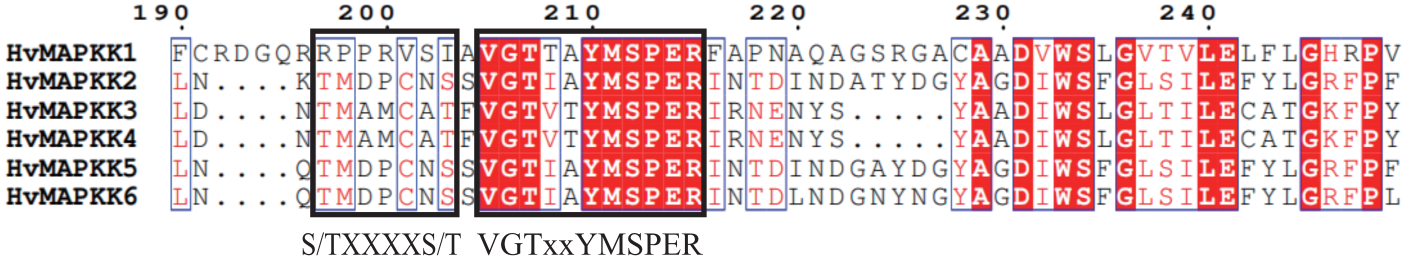
190
200
210
220
230
240
HvMAPKK1 FCRDGQRRPPRVSIAVGTTAYMSPERFAPNAQAGSRGACAADVWSLGVTVLELFLGHRPV
HvMAPKK2 LN....KTMDPCNSSVGTIAYMSPERINTDINDATYDGYAGDIWSFGLSILEFYLGRFPF
HvMAPKK3 LD....NTMAMCATFVGTVTYMSPERIRNENYS.....YAADIWSLGLTILECATGKFPY
HvMAPKK4 LD....NTMAMCATFVGTVTYMSPERIRNENYS.....YAADIWSLGLTILECATGKFPY
HvMAPKK5 LN....QTMDPCNSSVGTIAYMSPERINTDINDGAYDGYAGDIWSFGLSILEFYLGRFPF
HvMAPKK6 LN....QTMDPCNSSVGTIAYMSPERINTDLNDGNYNGYAGDIWSFGLSILEFYLGRFPL
S/TXXXXS/T
VGTxxYMSPER
