# Supplementary material for: Genome-wide identification, expression profiles and regulatory network of MAPK cascade gene family in barley
Source: BMC Genomics. 2019 Oct 17;20:750. doi: 10.1186/s12864-019-6144-9 (PMC6796406; doi:10.1186/s12864-019-6144-9)
Supplement: Supplementary file 4 — Additional file 4: Figure S4. Multiple sequence alignment of the HvMAPKKK to identify the conserved kinase motifs. The red color marked are the signature motif of MEKK, Raf and ZIK three sub family. [file 12864_2019_6144_MOESM4_ESM.pdf]

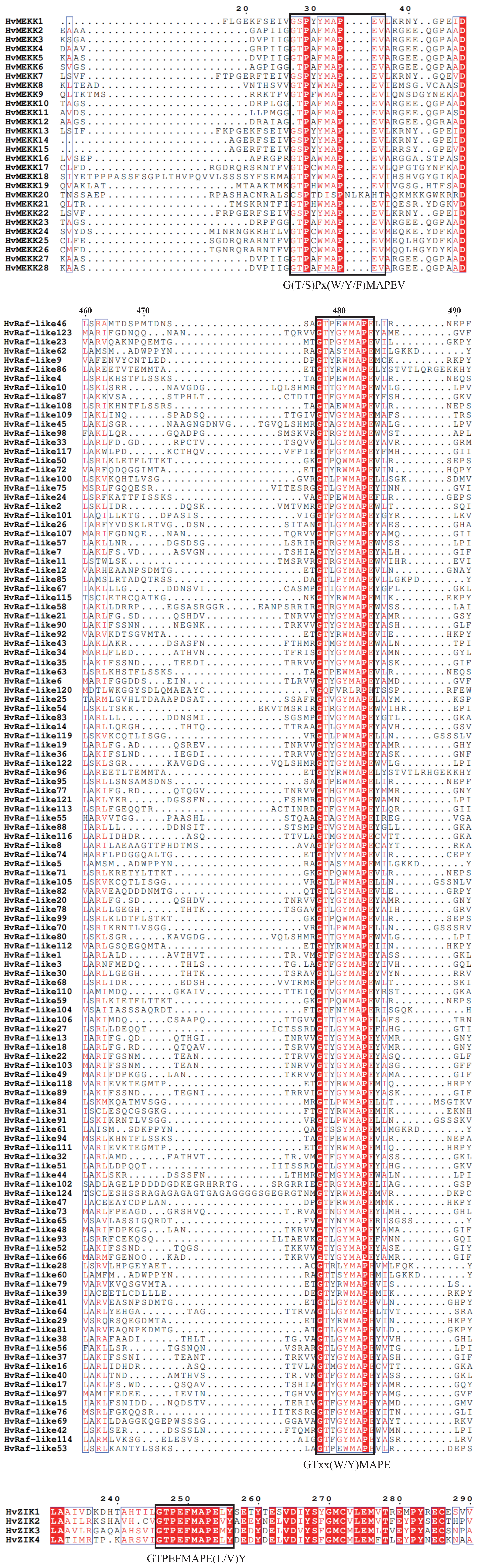

G(T/S)Px(W/Y/F)MAPEV
GTxx(W/Y)MAPE
GTPEFMAPE(L/V)Y
