# Supplementary figures and images for: Genome-wide identification, expression profiles and regulatory network of MAPK cascade gene family in barley
Source: BMC Genomics. 2019 Oct 17;20:750. doi: 10.1186/s12864-019-6144-9 (PMC6796406; doi:10.1186/s12864-019-6144-9)

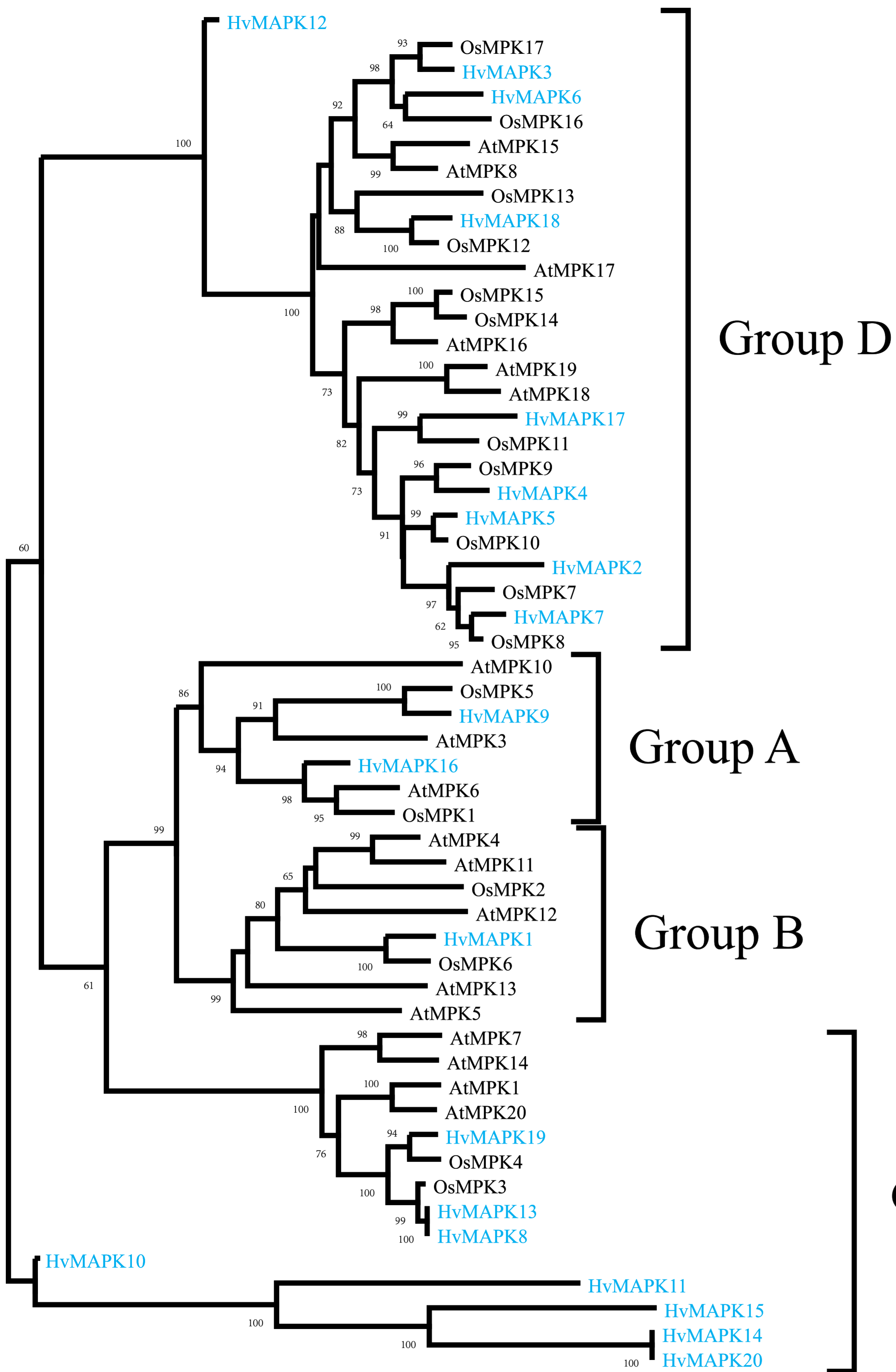

20

Supplement: Supplementary file 5 — Additional file 5: Figure S5. Evolutionary relationships and grouping among barley, rice and Arabidopsis MAPKs. [file 12864_2019_6144_MOESM5_ESM.pdf]

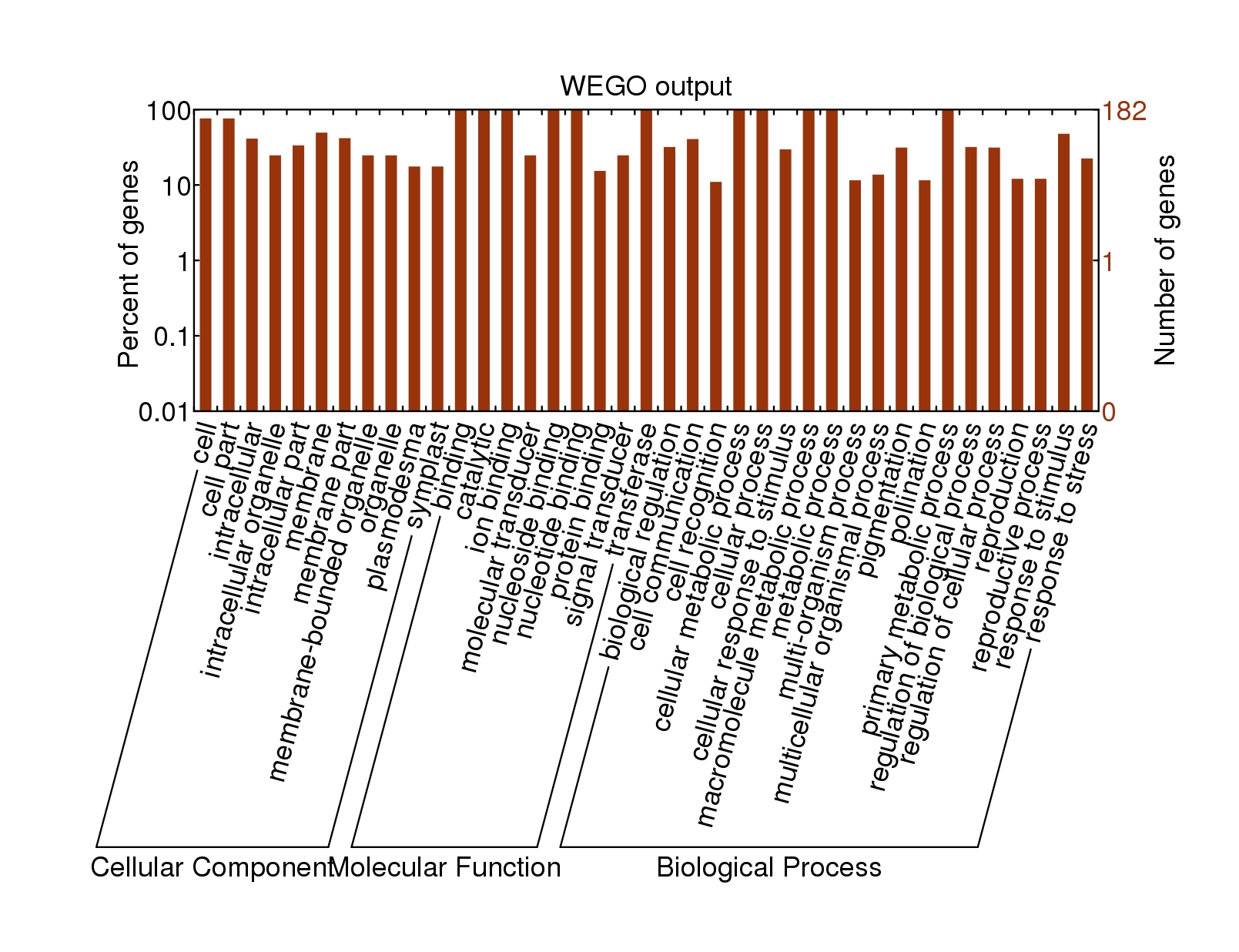

Supplement: Supplementary file 6 — Additional file 6: Figure S6. GO annotation of these identified barley MAPK cascade genes. [file 12864_2019_6144_MOESM6_ESM.jpg]
